# The Bacterial Community in Questing Ticks From Khao Yai National Park in Thailand

**DOI:** 10.3389/fvets.2021.764763

**Published:** 2021-11-22

**Authors:** Ratree Takhampunya, Jira Sakolvaree, Nitima Chanarat, Nittayaphon Youngdech, Kritsawan Phonjatturas, Sommai Promsathaporn, Bousaraporn Tippayachai, Wirunya Tachavarong, Kanchit Srinoppawan, Betty K. Poole-Smith, P. Wesley McCardle, Suwanna Chaorattanakawee

**Affiliations:** ^1^Department of Entomology, Armed Forces Research Institute of Medical Sciences-United States Army Medical Directorate, Bangkok, Thailand; ^2^Department of Parasitology and Entomology, Faculty of Public Health, Mahidol University, Bangkok, Thailand; ^3^Department of National Parks, Wildlife and Plant Conservation, Bangkok, Thailand

**Keywords:** co-infection, metagenomics in ticks, *Amblyomma* spp., *Haemaphysalis* spp., *Dermacentor* spp., questing ticks in Thailand

## Abstract

Ticks are known vectors for a variety of pathogens including bacteria, viruses, fungi, and parasites. In this study, bacterial communities were investigated in active life stages of three tick genera (*Haemaphysalis, Dermacentor*, and *Amblyomma*) collected from Khao Yai National Park in Thailand. Four hundred and thirty-three questing ticks were selected for pathogen detection individually using real-time PCR assays, and 58 of these were subjected to further metagenomics analysis. A total of 62 ticks were found to be infected with pathogenic bacteria, for a 14.3% prevalence rate, with *Amblyomma* spp. exhibiting the highest infection rate (20.5%), followed by *Haemaphysalis* spp. (14.5%) and *Dermacentor* spp. (8.6%). *Rickettsia* spp. were the most prevalent bacteria (7.9%) found, followed by *Ehrlichia* spp. (3.2%), and *Anaplasma* spp. and *Borrelia* spp. each with a similar prevalence of 1.6%. Co-infection between pathogenic bacteria was only detected in three *Haemaphysalis* females, and all co-infections were between *Rickettsia* spp. and Anaplasmataceae (*Ehrlichia* spp. or *Anaplasma* spp.), accounting for 4.6% of infected ticks or 0.7% of all examined questing ticks. The prevalence of the *Coxiella*-like endosymbiont was also investigated. Of ticks tested, 65.8% were positive for the *Coxiella*-like endosymbiont, with the highest infection rate in nymphs (86.7%), followed by females (83.4%). Among tick genera, *Haemaphysalis* exhibited the highest prevalence of infection with the *Coxiella*-like endosymbiont. Ticks harboring the *Coxiella*-like endosymbiont were more likely to be infected with *Ehrlichia* spp. or *Rickettsia* spp. than those without, with statistical significance for *Ehrlichia* spp. infection in particular (*p*-values = 0.003 and 0.917 for *Ehrlichia* spp. and *Rickettsia* spp., respectively). Profiling the bacterial community in ticks using metagenomics revealed distinct, predominant bacterial taxa in tick genera. Alpha and beta diversities analyses showed that the bacterial community diversity and composition in *Haemaphysalis* spp. was significantly different from *Amblyomma* spp. However, when examining bacterial diversity among tick life stages (larva, nymph, and adult) in *Haemaphysalis* spp., no significant difference among life stages was detected. These results provide valuable information on the bacterial community composition and co-infection rates in questing ticks in Thailand, with implications for animal and human health.

## Introduction

Ticks are recognized as a medically important group of arthropods that transmit a number of diseases to humans (1). Different tick species favor different habitats, which ultimately defines their geographical distribution and thus the risk areas for human or animal infections. Several pathogens are known to be carried by either hard ticks (Ixodidae) or soft ticks (Argasidae), including a wide range of viruses, bacteria, fungi, and protozoa (2–6).

In Southeast Asia, 97 tick species (5 in Argasidae and 93 in Ixodidae) have been described (7). In Thailand, four species within Argasidae (2 *Argas* and 2 *Ornithodoros*) and 58 species within Ixodidae have been recorded. Of the hard ticks in Thailand, there are 25 species within genus *Haemaphysalis*, 10 *Ixodes*, 13 *Amblyomma*, 5 *Dermacentor*, 4 *Rhipicephalus*, and *Nosomma monstrosum*. Among tick species found in Thailand, *Amblyomma* spp. and *Dermacentor* spp. were associated with human otoacariasis, especially *Amblyomma testudinarium* (8) and *Dermacentor steini* (9). However, *Haemaphysalis* spp. and other tick genera are occasionally found on humans as well. *Rickettsia* spp. are the main tick-borne pathogens causing human infections in Thailand (10–12) with scattered reports of other tick-borne disease (TBD) in human and animals such as Q fever (13, 14) and anaplasmosis in companion pets and animals (15–17).

Ticks also harbor many non-pathogenic organisms. Bacterial endosymbionts have been recognized as important microorganisms required for tick fitness, especially with regard to regulating host reproduction and immunity (18, 19). Some studies suggest that these symbionts have a potential role in providing key vitamins absent in the bloodmeal (20, 21). Others reported that they might have an important role in facilitating pathogen colonization in the gut as in the case in *Ixodes scapularis* ticks, in which alteration of the symbiont abundance resulted in decreased *Borrelia burgdorferi* colonization (22). Likewise, the level of *Anaplasma marginale* acquisition was lower in *Dermacentor andersoni* when their microbiome was altered by antibiotic treatment leading to an increase in proportion and quantity of *Rickettsia bellii* in the microbiome (23). Moreover, the prevalence and transovarial transmission of bacterial endosymbionts occurs at a high rate, suggesting that they might have an obligate relationship with the host (24, 25). In addition to the bacterial microbiota in ticks, other microorganisms are as important and abundant. For example, several studies reported that two groups of bunyaviruses (South Bay virus and Phlebovirus) were commonly associated with *Ixodes* spp. ticks in America and Europe (26–28) and were more abundant than bacteria or eukaryote counterparts (29).

It is thought that because ticks harbor a diverse range of microorganisms, co-infection or co-occurrence of bacteria, parasites, and viruses in ticks is possible (30). Co-infection may lead to increased disease severity as it complicates disease diagnosis and treatment (31–34). Co-infections between *B. burgdorferi* and *Anaplasma phagocytophilum* are widely recognized and reported (35). Other co-infections among different types of microorganisms have also been reported, such as between *B. burgdorferi* and South Bay virus (SBV), *Babesia microti*, and *B. burgdorferi*, as well as between a novel filarial worm (*Onchocercidae* sp. ex. *Ixodes scapularis*) and *Wolbachia* spp (29).

In this study, we used both conventional and high-throughput sequencing methods to study bacterial pathogen co-infections and pathogen association with bacterial endosymbionts in questing ticks collected in Khao Yai National Park. Metagenomics were used to determine the bacterial communities in individual ticks and conventional methods (real-time PCR, PCR, and Sanger sequencing) were used to detect pathogenic bacteria: *Rickettsia* spp., *Anaplasma* spp., *Ehrlichia* spp., *Borrelia* spp., *Coxiella burnetii*, and pathogenic *Francisella* spp. in *Amblyomma* spp., *Dermacentor* spp., and *Haemaphysalis* spp. ticks individually. All positive samples underwent DNA sequencing to identify pathogens to the species level. Co-infections between bacteria and the association between pathogenic bacteria and the *Coxiella*-like endosymbiont were investigated.

## Materials and Methods

### Tick Collection

Questing ticks were collected by dragging a 1-m^2^ cotton cloth over vegetation in four tourist attraction sites in Khoa Yai National Park (14°26'19.5“N 101°22'20.1”E). Sampling was conducted by six people at each site for 1 h. Ticks were collected in one trip in November 2020 and immediately preserved in 90% ethanol before transporting to the laboratory for further processing. All sampling procedures and experimental manipulations were reviewed and approved as part of the animal collection protocol entitled “Surveillance of Tick- and Flea-Borne Diseases of Public Health Importance” (PN# 21-01). The project was also approved by Mahidol University – Institute animal care and use committee (MU-IACUC 2019/3). Research was conducted in compliance with the Animal Welfare Act and other federal statutes and regulations related to animals and experiments involving animals, and adhered to principles outlined in the “Guide for the Care and Use of Laboratory Animals,” NRC Publication, 2011 edition.

### Tick Surface Sterilization and DNA Extraction

Questing ticks (unfed ticks) were morphologically identified using taxonomic keys (36, 37). Each tick was vortexed for 1 min in 3% sodium hypochlorite and then transferred to a new tube and vortexed for 1 min in 70% alcohol followed by three washes in sterile PBS in the same manner. Ticks were air dried for 10 min on Whatman® filter paper (Sigma-Aldrich, Saint Louis, MO, USA) before DNA extraction. Whole ticks in 250 μl of ATL buffer (lysis buffer, component of the extraction kit) were punctured with a fine tip under a stereomicroscope to release the tissue from the hard chitin exoskeleton prior to adding 2 mg/ml of Proteinase K solution. Samples were then incubated at 55°C overnight. A total volume of 250 μl of homogenized solution was then used for DNA extraction on the QIAsymphony® SP instrument with QIAsymphony® DSP DNA Mini Kit using Tissue LC 200 DSP protocol (Qiagen, Hombrechtikon, Switzerland). The DNA was eluted in 50 μl of ATE buffer (elution buffer, component of extraction kit) and stored at−20°C until use. Ultrapure DNA/RNA-free distilled water as well as PBS buffer used for tick surface sterilization were also included as an extraction control.

### Amplification of Bacterial 16S rDNA

Following DNA extraction, the bacterial-specific 16S rDNA (V3–V4, a 550-bp fragment) was amplified as previously described (15). Negative control PCR reactions were included in every experimental run using Ultrapure DNA/RNA-free distilled water in place of DNA template. PCR reactions were also performed with eluates from mock DNA extractions as well as from PBS buffer used for tick surface sterilization. PCR product was cleaned using AMPure magnetic bead-based purification system (Beckman Coulter, UK) following the manufacturer's instructions. Purified PCR products were eluted and quantified using the Qubit dsDNA HS Assay Kit (Invitrogen Life Technologies, MA).

### Library Preparation and High-Throughput Sequencing

The library was prepared with dual indices and Illumina sequencing adapters attached to purified PCR products using the Nextera XT Index Kit following the manufacturer's protocol (Illumina Inc., San Diego, CA). For index control reaction, a combination of index primers that were not used with samples was also included with PCR grade water as template. The number of reads recovered from these particular index combinations were used to filter the cross-contaminations between indexed PCR primers and to identify errors in an Illumina sample sheet. Libraries were cleaned using Agencourt AMPure XP beads. The purity of the libraries was checked on the QIAxcel Advanced System (Qiagen) with a QIAxcel DNA High Resolution Cartridge. Purified amplicon libraries were quantified using the Qubit dsDNA HS Assay Kit (Invitrogen). DNA concentration was calculated and normalized to reach 4.0 nM for each library. Five microliters of DNA from each library was pooled for a NGS run. Pooled libraries were denatured and diluted to a final concentration of 8 pM with a 10% PhiX (Illumina) control. Sequencing was performed using the MiSeq Reagent Kit V3 on the Illumina MiSeq System.

### Data Analysis for Metagenomics and Diversity Estimates

The sequence reads generated by the 16S rRNA on MiSeq sequencers were processed on CLC Genomics workbench v 12.0.3 (Qiagen, Aarhus A/S, http://www.clcbio.com). High-throughput sequences were imported into CLC Genomics Workbench according to quality scores of Illumina pipeline 1.8. In order to achieve the highest quality sequences for clustering, paired reads were merged in CLC microbial genomics module v 4.8 using default settings (mismatch cost = 1; minimum score = 40; gap cost = 4 and maximum unaligned end mismatch = 5). Primer sequences were trimmed from merged reads using parameters (trim using quality scores = 0.01, trim ambiguous nucleotides = 2, and discard read length shorter than 150 bp). Samples were removed from analysis if the number of reads was <100 or <25% from the median (the median number of reads across all samples). Chimeric sequences were detected and removed. Only filtered and merged sequences were clustered into operational taxonomic units (OTUs) according to a threshold of 97% sequence identity. All such processes were performed using CLC microbial genomics module v 4.8. Reference OTU data used in the present study were downloaded from the Greengenes database V13.8 (38). OTUs with combined abundance <10 reads were removed from the downstream analysis. Alpha diversity estimates (Observed OTUs, Simpson's index, and Shannon entropy) were analyzed on the quality-filtered OTU table at the genus level using CLC Microbial Genomics Module v 4.8. Beta diversity analysis for microbiome compositional difference between groups was calculated using a distance-based non-parametric test, the generalized UniFrac distances (39). The statistical significance of microbiome compositional difference between groups (tick genera, tick developmental stages, *Francisella persica* infection, *Coxiella*-like endosymbiont infection, and *Rickettsia* spp. infection statuses) was then compared using PERMANOVA using 99,999 permutations of the distance values. All analyses mentioned here were performed with CLC Microbial Genomics Module v 4.8.

### Bacterial Pathogen Detection by Real-Time PCR and Conventional PCR

Real-time PCR and PCR assays were performed on 433 individual ticks for the detection of bacteria (*Rickettsia* spp., *Borrelia* spp., *Anaplasma* spp., *Ehrlichia* spp., and *Coxiella*-like endosymbiont) and the taxonomic species assignment. Other potential pathogenic bacteria (*Francisella* spp. and *Coxiella* spp.) detected by NGS analysis (read count > 1) were also confirmed by real-time PCR and PCR assays. Detailed methods for assays and target gene(s) for selected pathogens are provided as online Supplementary data (Supplementary Table 1). For all real-time PCR, the reaction consisted of 1X Platinum quantitative PCR SuperMix-UDG (Invitrogen) using standard real-time PCR conditions with primer/probe concentrations and annealing temperatures as indicated in Supplementary Table 1. For conventional PCR, the assay was carried out in a 50-μl reaction volume containing 0.5 U of iProof High-Fidelity DNA Polymerase, 200 μM dNTPs, MgCl_2_, and primer concentration as indicated (Supplementary Table 1). The PCR conditions consisted of 98°C for 3 min, followed by 40 cycles of 98°C for 10 s, annealing temperature for 30 s (indicated in Supplementary Table 1 for each pathogen), and 72°C for 45 s.

### DNA Sequencing

PCR amplicons were purified using the QIAquick® PCR Purification Kit (Qiagen) according to the manufacturer's instructions. The PCR products were cycle-sequenced using an ABI BigDye™ Terminator v3.1 Cycle Sequencing Kit, ethanol precipitated, and run on a SeqStudio Genetic Analyzer (Applied Biosystems ThermoFisher, Thailand). Sequences of each sample and pathogen were assembled using Sequencher™ ver. 5.4.6 (GeneCodes Corp., Ann Arbor, MI). The pathogen sequences were aligned with reference sequences retrieved from the GenBank database using the MUSCLE codon alignment algorithm (40). A maximum likelihood phylogenetic tree was then constructed from bacterial target genes (Supplementary Table 1) using the best fit model of nucleotide substitution with bootstrapping (1,000 replicates) in MEGA 6 (41).

### Statistical Analysis

Differences in alpha diversity indices of the bacterial community composition, based on metagenomics data, were determined by Mann–Whitney *U* test (between two groups) or Kruskal–Wallis test (across all groups) and the critical range (*p* < 0.05) was determined. Statistical analyses (two-way ANOVA tests and mean, 95% confidence interval) and scatter plots were performed using GraphPad Prism version 5.04 for Windows (GraphPad Software, San Diego, CA. www.graphpad.com). Some graphical illustrations presented in this study as well as the Chi-square independence test and Fisher's exact test were performed in the R environment for statistical computing (42, 43). A nucleotide distance matrix was generated using “Compute Pairwise Distance” in MEGA 6 (41).

## Results

### Tick Species Diversity

A total of 21,229 questing ticks were collected in November 2020 in Khao Yai National Park. The majority of ticks collected were larvae (*n* = 20,916) accounting for 98.5%, followed by females (*n* = 145, 0.7%), males (*n* = 85, 0.4%), and nymphs (*n* = 83, 0.4%). Species identification was done for the adult stage only and *Haemaphysalis lagrangei, Haemaphysalis obesa*, and *Haemaphysalis longicornis* were found at the highest rate (22%−24%), followed by *Haemaphysalis shimoga* (15%), *Dermacentor auratus* (9%), *Amblyomma testudinarium* (4%), *Haemaphysalis papuana* (1%), and *Dermacentor steini* (1%). All adult ticks (*n* = 230) and nymphs (*n* = 83) and 0.6% of larvae (*n* = 120) were selected for pathogen detection individually (*n* = 433). Identification of larval stage was performed on 120 selected samples and *Amblyomma* spp. (*n* = 35), *Dermacentor* spp. (*n* = 35), and *Haemaphysalis* spp. (*n* = 50) were found. In total, there were *Haemaphysalis* spp. (331, 76.4%), *Dermacentor* spp. (58, 13.4%), and *Amblyomma* spp. (44, 10.2%) included in this study. Additionally, a subset of ticks (*n* = 82) were selected for studying the bacterial community profile using 16S rRNA Next-Generation Sequencing (Table 1). Five to six ticks per species and life stage were selected for NGS, with the exception of immature *Haemaphysalis* spp., where 10 larvae and 20 nymphs were included. The number of ticks selected for NGS and the final number of ticks that passed the quality filter are described in detail in the online Supplementary data (Supplementary Table 2).

**Table 1:** Tick species collected in Khao Yai National Park, Thailand (2020).

**Tick species**	**Larvae**	**Nymphs**	**Males**	**Females**	**Selected for NGS**	**Passed quality-filter**
Unidentified Larvae	20,796	0	0	0	0	0
*Amblyomma* spp.	35	2	0	0	7	7
*Amblyomma testudinarium*	0	0	1	6	4	1
*Dermacentor* spp.	35	0	0	0	5	5
*Dermacentor auratus*	0	0	6	14	6	4
*Dermacentor steini*	0	0	1	2	3	1
*Haemaphysalis* spp.	50	0	0	0	10	9
*Haemaphysalis lagrangei*	0	0	4	51	6	3
*Haemaphysalis longicornis*	0	0	34	17	6	5
*Haemaphysalis obesa*	0	0	26	30	6	3
*Haemaphysalis papuana*	0	0	0	3	3	1
*Haemaphysalis shimoga*	0	0	13	22	6	4
*Haemaphysalis* spp.	0	81	0	0	20	15
Total	20,916	83	85	145	82	58
Selected for study	120^*^	83	85	145	82	58

*Number of ticks included in this study and for studying bacterial community profile using 16S rRNA gene Next-Generation Sequencing is shown.*

(*), only larvae belonging to the three tick genera were selected for further analyses.

### Pathogen Prevalence in Questing Ticks and Species Identification

**Figure 1:**
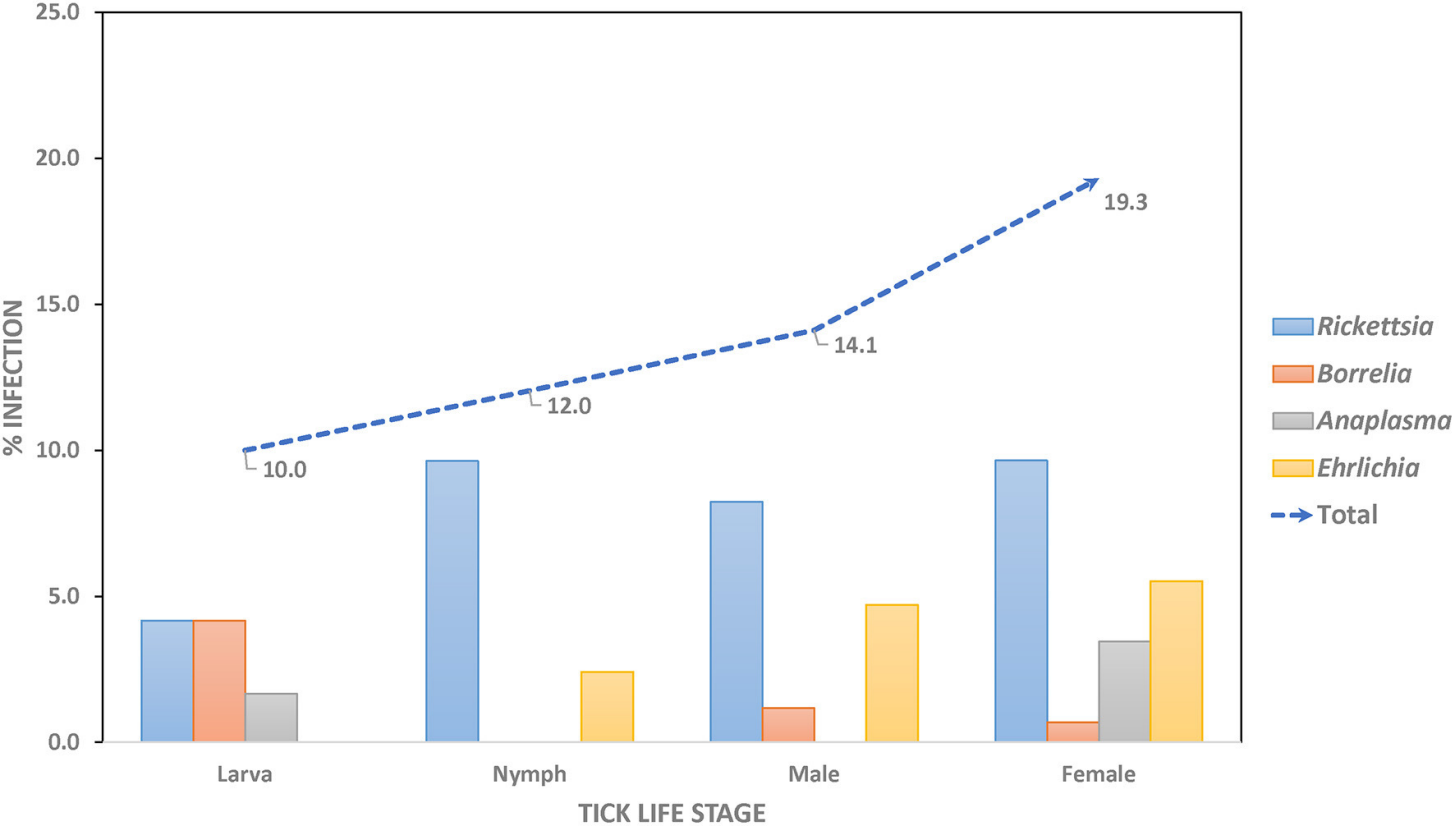
Prevalence rate of pathogenic bacteria in questing ticks, by stages.

Overall, pathogenic bacteria were detected in 62 out of the total number of examined ticks collected from Khao Yai National Park, for a 14.3% prevalence rate. Pathogens were found in 48 of the tested *Haemaphysalis* spp. (14.5%), nine in *Amblyomma* spp. (20.5%), and five in *Dermacentor* spp. (8.6%). *Rickettsia* spp. was the most common pathogenic bacteria (7.9%), followed by *Ehrlichia* spp. (3.2%), and *Anaplasma* spp. and *Borrelia* spp. with a similar rate of 1.6% (Table 2). *Haemaphysalis* spp. (*n* = 331) were infected by all pathogens mentioned with *Rickettsia* spp. at the highest rate (7.6%), followed by *Ehrlichia* spp. (3.2%), *Anaplasma* spp. (1.8%), and *Borrelia* spp. (0.9%). In *Amblyomma* spp. (*n* = 44) and *Dermacentor* spp. (*n* = 58), two bacterial pathogens were found in each genus. Both were infected with *Rickettsia* (18.2% and 1.7% prevalence rates, respectively). *Anaplasma* spp. was detected only in *Amblyomma* spp. (2.3%), while *Borrelia* spp. was detected only in *Dermacentor* spp. (6.9%). When examining pathogen prevalence in tick life stages, the infection rate was found to increase from 10.0 to 19.3% from larvae to adults, respectively (Figure 1). The trend of increasing pathogen prevalence by life stage is clearly observed for *Rickettsia* spp. and *Ehrlichia* spp.

**Table 2:** Prevalence of pathogenic bacteria (*Rickettsia* spp., *Anaplasma* spp., *Ehrlichia* spp., and *Borrelia* spp.) in questing ticks from Khao Yai National Park, by species and stages.

**Species**	**Stages**	***N***	**No. of positive (% Infection)**				
			**All pathogens**	***Rickettsia*** **spp**.	***Anaplasma*** **spp**.	***Ehrlichia*** **spp**.	***Borrelia*** **spp**.
*Amblyomma* spp.	Immature	37	5 (13.5%)	4 (10.8%)	1 (2.7%)	0	0
*A. testudinarium*	Mature	7	4 (57.1%)	4 (57.1%)	0	0	0
Total	All stages	44	9 (20.5%)	8 (18.2%)	1 (2.3%)	0	0
*Dermacentor* spp.	Immature	35	4 (11.4%)	0	0	0	4 (11.4%)
All species	Mature	23	1 (4.3%)	1 (4.3%)	0	0	0
*D. steini*	Mature	3	1 (33.3%)	1 (33.3%)	0	0	0
*D. auratus*	Mature	20	0	0	0	0	0
Total	All stages	58	5 (8.6%)	1 (1.7%)	0	0	4 (6.9%)
*Haemaphysalis* spp.	Immature	131	13 (9.9%)	9 (6.9%)	1 (0.8%)	2 (1.5%)	1 (0.8%)
All species	Mature	200	35 (17.5%)	16 (8.0%)	5 (2.5%	12 (6.0%)	2 (1.0%)
*H. shimoga*	Mature	35	3 (8.6%)	3 (8.6%)	0	0	0
*H. longicornis*	Mature	51	10 (19.6%)	5 (9.8%)	1 (2.0%)	4 (7.8%)	0
*H. lagrangei*	Mature	55	14 (25.5%)	8 (14.5%)	1 (1.8%)	5 (9.1%)	0
*H. obesa*	Mature	56	8 (14.3%)	0	3 (5.4%)	3 (5.4%)	2 (3.6%)
*H. papuana*	Mature	3	0	0	0	0	0
Total	All stages	331	48 (14.5%)	25 (7.6%)	6 (1.8%)	14 (4.2%)	3 (0.9%)
Grand Total		433	62 (14.3%)	34 (7.9%)	7 (1.6%)	14 (3.2%)	7 (1.6%)

*Immature, larva and nymph; Mature, adult female and male*.

Sequence and phylogenetic analyses showed that among *Rickettsia* species detected in ticks, *R. montana* (*n* = 21) was the predominant species found in *Haemaphysalis* spp., while *R. raoultii* (*n* = 6) was detected mostly in *Amblyomma* spp. (*n* = 5), and three other *Rickettsia* species (2 = *R. heilongjiangensis*, 3 = *R. monacensis*, and 2 = *Rickettsia* sp.) were detected in *Haemaphysalis* spp. and *Amblyomma* spp. (Table 3). *Borrelia theileri* (*n* = 7) was the only *Borrelia* species found, and was found in nearly equal numbers in *Dermacentor* spp. and *Haemaphysalis* spp. ticks. The majority of Anaplasmataceae bacteria (7 = *Anaplasma* spp. and 14 = *Ehrlichia* spp.) were mostly detected in *Haemaphysalis* spp. with *E. ewingii* (*n* = 9) being the dominant species found. Phylogenetic analyses for all bacteria can be found in online Supplementary Data (Supplementary Figure 1).

**Table 3:** Bacterial species identification by DNA sequence and phylogenetic analyses.

	**No. of positive (% Sequence identity)**			
**Pathogen species**	***Amblyomma*** **spp**.	***Dermacentor*** **spp**.	***Haemaphysalis*** **spp**.	**Total**
*Rickettsia* sp.	0	0	2 (99.4%)	2
*Rickettsia raoultii*	6 (99.2–99.4%)	1^*^	0	7
*Rickettsia montana*	0	0	21 (98.8–99.0%)	21
*Rickettsia monacensis/Rickettsia tamurae*	3 (99.0–100%)	0	0	3
*Rickettsia heilongjiangensis*	0	0	1 (100%)	1
*Borrellia theileri*	0	4 (99.3–99.8%)	3 (99.8%)	7
*Anaplasma* sp.	1^*^	0	4 (99.6–100%)	5
*Anaplasma bovis*	0	0	2 (100%)	2
*Ehrlichia* spp. similar to *E. ewingii^#^*	0	0	4 (99.1–100%)	4
*Ehrlichia* spp. similar to *Ehrlichia ewingii*	0	0	9 (91.7–94.4%)	9
*Ehrlichia* sp. similar to *Candidatus* Ehrlichia shimanensis	0	0	1 (96.6%)	1
Total	9	5	48	62

(*), short sequence, species identification was from the most similar reference sequence from BLASTN search,

*(#), sequence similarity based on 16S rRNA gene*.

### Co-infection of Bacterial Pathogens

**Figure 2:**
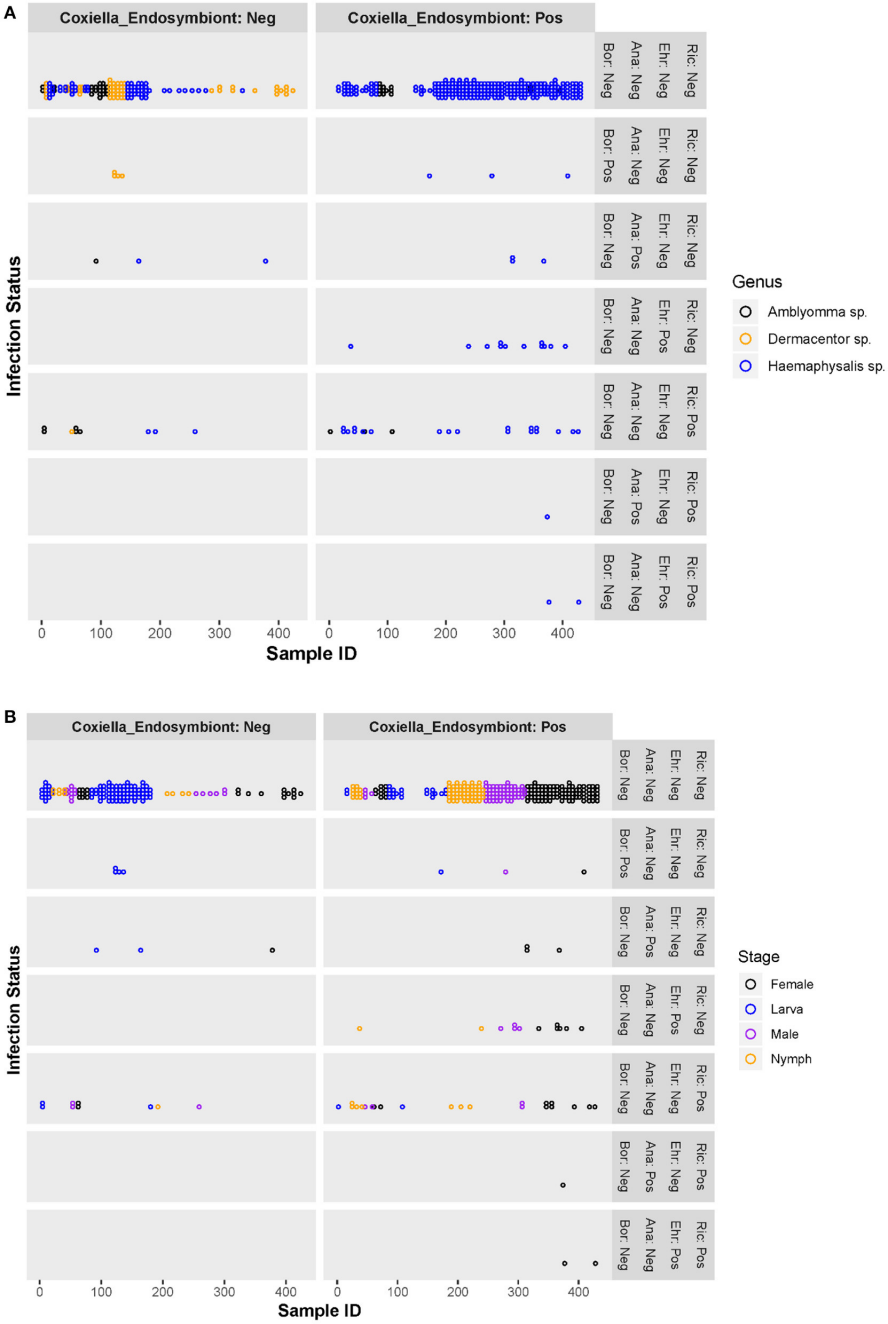
Scatter plots of individual ticks (with or without *Coxiella*-like endosymbiont) and their pathogenic bacterial infection status, by tick genera **(A)** and tick stages **(B)**. Ric, *Rickettsia* spp.; Ehr, *Ehrlichia* spp.; Ana, *Anaplasma* spp.; Bor, *Borrelia* spp.; Coxiella_Endosymbiont, *Coxiella*-like endosymbiont; Pos, positive; Neg, negative.

Co-infection between pathogenic bacteria examined in this study only occurred in a small number of ticks, primarily female *Haemaphysalis* spp. (*n* = 3). All co-infections occurred with *Rickettsia* spp. and Anaplasmataceae (*Ehrlichia* spp. or *Anaplasma* spp.) (Figures 2A,B) and accounted for 4.8% of infected ticks and 0.7% of all ticks examined. All three co-infected ticks were also positive for the *Coxiella*-like endosymbiont.

### Bacterial Endosymbionts and Co-occurrence With Pathogenic Bacteria

The *Coxiella*-like endosymbiont was detected in 65.8% of all ticks examined, with the highest infection rate in nymphs (86.7%) and females (83.4%) (Table 4). Of all genera, *Haemaphysalis* spp. exhibited the highest rate of infection with the *Coxiella*-like endosymbiont, with an 81.0% infection rate. The majority of *Haemaphysalis* spp. infected were females (95.1%), followed by nymphs (88.9%), males (87.0%), and, to a lesser extent, larvae (24.0%). The second highest infection rate belonged to *Amblyomma* spp. with a 38.6% infection rate and only detected in females (66.7%) and larvae (37.1%). Surprisingly, no *Dermacentor* spp. were positive for the *Coxiella*-like endosymbiont.

**Table 4:** Prevalence of *Coxiella*-like endosymbiont in questing ticks, by tick genera and stages.

**Tick species**	**Total number of tick tested (*N*)**	**No. of *Coxiella*-like endosymbiont-positive ticks/total no. of tick (% Infection)**				
		**Larva**	**Nymph**	**Male**	**Female**	**All stages**
*Amblyomma* spp.	44	13/35 (37.1)	0/2 (0)	0/1 (0)	4/6 (66.7)	17/44 (38.6)
*Dermacentor* spp.	58	0/35 (0)	0/0 (0)	0/7 (0)	0/16 (0)	0/58 (0)
*Haemaphysalis* spp.	331	12/50 (24.0)	72/81 (88.9)	67/77 (87.0)	117/123 (95.1)	268/331 (81.0)
Total	433	25/120 (20.8)	72/83 (86.7)	67/85 (78.8)	121/145 (83.4)	285/433 (65.8)

Figures 2A,B are scatter plots of individual ticks depicting the pathogenic bacterial infection status compared between two tick populations—those harboring the *Coxiella*-like endosymbiont and those without. There was no significant difference in the number of ticks infected with *Borrelia* spp. or *Anaplasma* spp. between the *Coxiella*-like endosymbiont-positive and -negative groups. However, ticks harboring the *Coxiella*-like endosymbiont had greater rates of *Ehrlichia* spp. or *Rickettsia* spp. infection than those without the *Coxiella*-like endosymbiont. Chi-square and Fisher's exact tests were used to test for significant differences of *Rickettsia* spp. or *Ehrlichia* spp. infection between endosymbiont-positive and -negative ticks. Results show that the proportion of *Rickettsia* spp. infection in ticks harboring the *Coxiella*-like endosymbiont [8.8%, CI (5.5, 12.1%)] was not significantly different from the proportion in ticks without the *Coxiella*-like endosymbiont [6.1%%, CI (2.2, 9.9%)] with statistical values; Chi-square = 0.638, df = 1, *p*-value = 0.424. Likewise, the proportion of all pathogenic bacterial infection between ticks harboring the *Coxiella*-like endosymbiont [16.1%, CI (11.9, 20.4%)] and ticks without *Coxiella*-like endosymbiont [10.8%, CI (5.8, 15.8%)] was not significantly different (Chi-square = 0.7374, df = 1, *p*-value = 0.3905). *Ehrlichia* infection was observed only in ticks harboring *Coxiella*-like endosymbiont with the proportion of 4.9%, CI [2.4%, 7.4%]. Fisher's Exact test was used to determine the association between *Ehrlichia* infection and infection with the *Coxiella*-like endosymbiont. Results indicate that infection by these two bacteria in ticks is dependent (alpha = 0.05, *p*-value = 0.003), suggesting that the *Ehrlichia* infection is significantly associated with ticks harboring the *Coxiella*-like endosymbiont.

### Bacterial Profile in Ticks

Of 433 samples tested in this study, 82 samples were selected for metagenomics NGS analysis (Table 1). After performing sequence quality filters and removing samples with low reads, only 58 samples (range: 1,085–472,236, mean number of reads ± SD = 37,780 ± 98,225) passed the criteria and were subjected to further OTU clustering and alpha and beta diversity analyses. A total of 2,222,970 reads passed quality filters and 268 OTUs were found across all samples. Comparison of the number of passed-filter reads being used for OTU clustering between tick stages showed that larvae (*n* = 19; 96,713 ± 157,880) generated more reads than nymphs (*n* = 17; 10,161 ± 9,845) and adults (*n* = 22; 8,226 ± 8,603). Eight *Amblyomma* spp., 10 *Dermacentor* spp., and 40 *Haemaphysalis* spp. were included in metagenomics analysis (Table 1).

**Figure 3:**
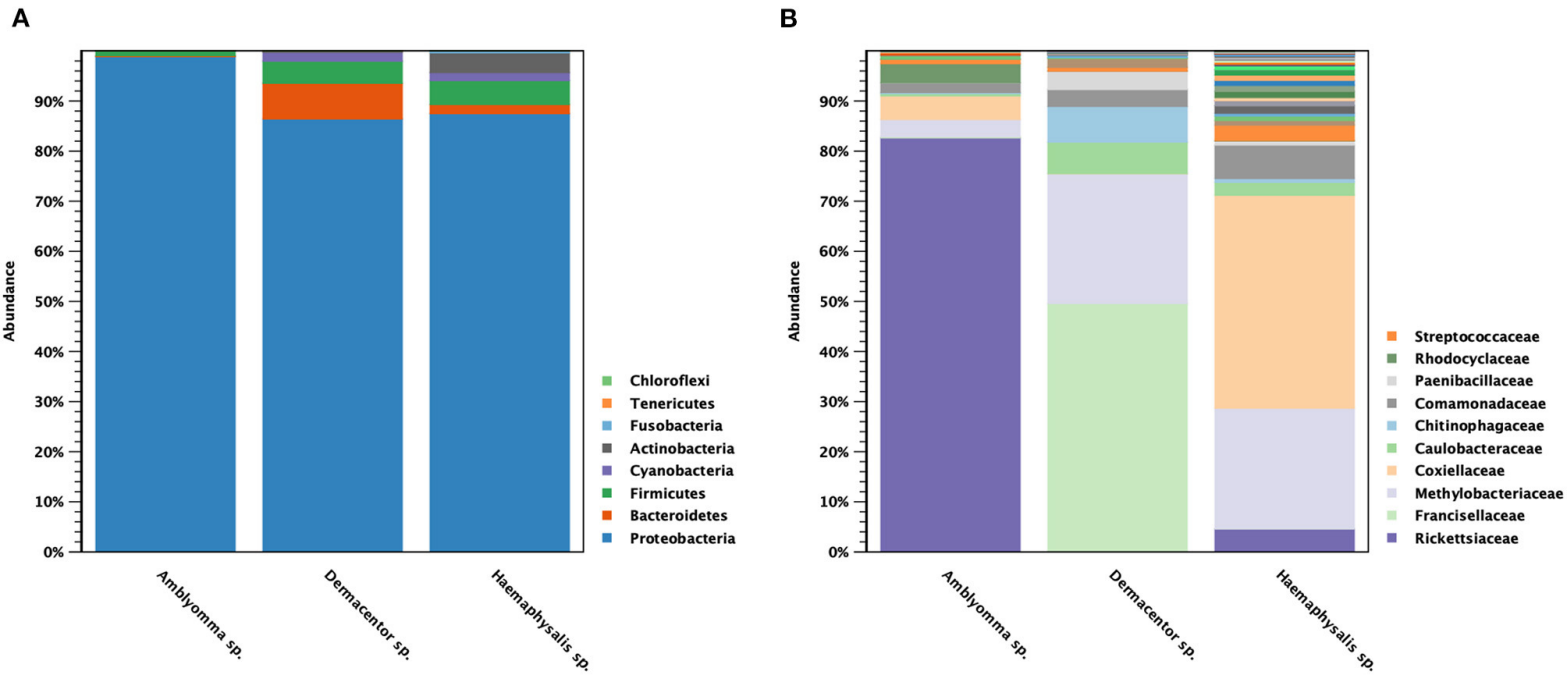
Taxonomic diversity and relative abundance at the phylum **(A)** and family **(B)** level of bacterial community in questing ticks. The percent relative abundances are of the total number of OTUs. Color legend for each phylum **(A)** or family **(B)** was indicated next to the bar graph.

The classification of OTUs from each sample was made against the Greengenes reference database and the similarity threshold was set at 0.97 in the CLC microbial genomics module. There were nine recorded phyla found among all tick samples studied: Proteobacteria (91%), Bacteroidetes (4%), Firmicutes (3%), Cyanobacteria (1%), and Actinobacteria (1%) (Figure 3A). There were five major phyla found in controls for DNA extraction, PCR, and Indexing; however, the majority of Proteobacteria phylum (73%) detected was genus *Enterobacteria* (89%), and very small amount of genus *Rickettsia* (0.0092%) (Supplementary Figure 2). Only 1% of reads could not be classified using Greengenes reference and were removed before performing downstream process of abundance analysis. Figure 3B shows the abundance of bacterial taxa in tick genera at the family level. There were one or two predominant bacterial taxa in each tick genus. Rickettsiaceae (83%) was the predominant bacterial taxon in *Amblyomma* spp., while Francisellaceae (50%) and Methylobacteriaceae (26%) were the major taxa found in *Dermacentor* spp. *Haemaphysalis* spp. harbored a more diverse bacterial spectrum than the other two tick genera. Coxiellaceae (43%) and Methylobacteriaceae (24%) were the predominant bacterial taxa found, with a lesser abundance of Rickettsiaceae (4.6%). Beta analysis using distance-based non-parametric test indicated that microbiome composition differed significantly across tick genera by the generalized UniFrac distance (df = 2, pseudo-*F* = 5.812, *p*-value = 0.00001).

**Figure 4:**
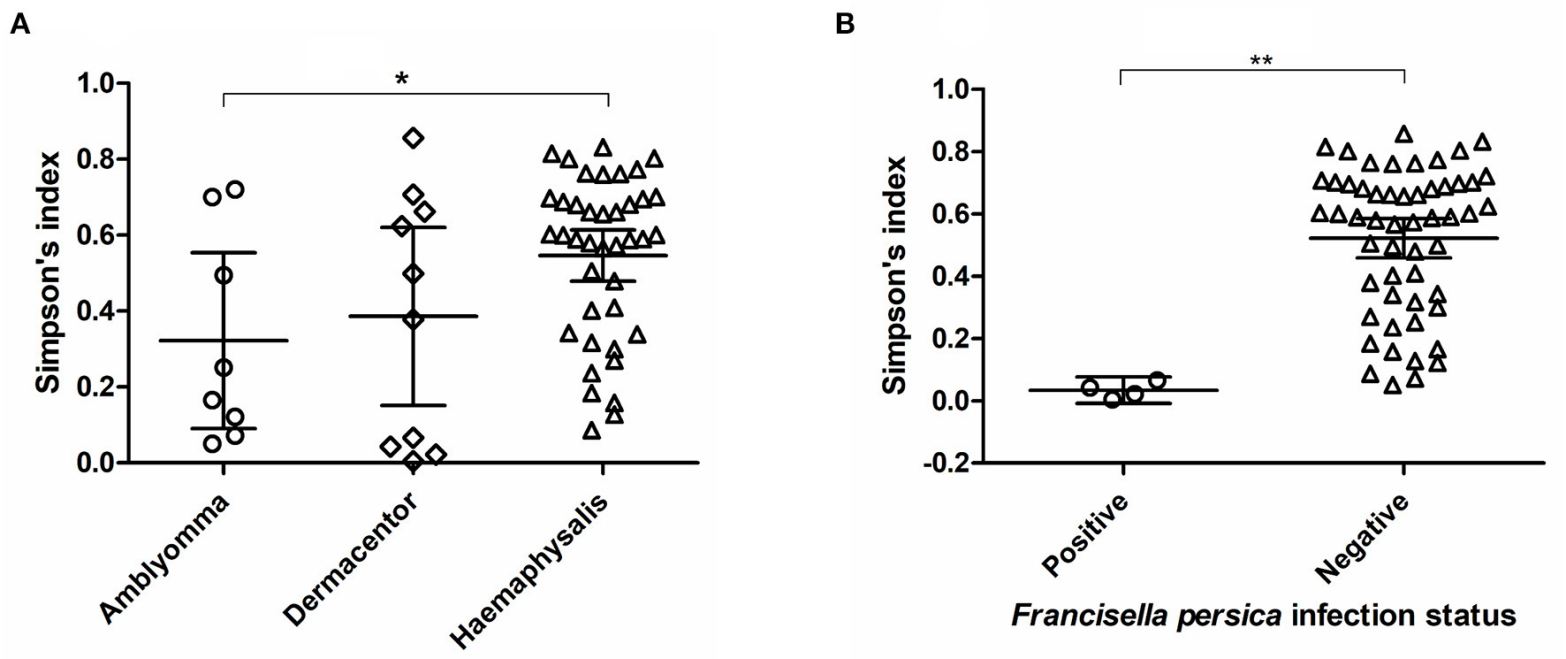
Alpha diversity measures (Simpson's index) based on 16S rRNA gene database for each tick genus **(A)** and *Francisella persica* infection status **(B)**. The statistically significant differences between groups are indicated (^*^p-value < 0.05, ^**^p-value < 0.001). The solid lines show mean and 95% confidence interval (CI) of alpha diversity for each group. Scatter plots and Mann–Whitney *U* test were created and tested using GraphPad Prism version 5.04.

Alpha diversity estimates of bacterial communities in ticks and statistical significance (*p*-value) for group comparison are summarized in Table 5. The alpha diversity analyses showed that the bacterial community of *Haemaphysalis* spp. (*n* = 40) was significantly different from *Amblyomma* spp. (*n* = 8) with *p*-value < 0.05 for all tests (Observed OTUs, Simpson's index, Shannon entropy) when measured at the genus level as shown in Figure 4A. While there were few ticks positive for *Francisella persica* (*n* = 4), alpha diversity estimates indicated that their bacterial profiles were quite different from ticks that were not infected with *F. persica* with statistical significance (*p*-value < 0.001) (Figure 4B).

**Table 5:** Alpha diversity estimates of bacterial communities based on metagenomics.

**Categories**	**Number**	**Diversity Mean [95% CI]**		
		**Observed OTUs**	**Simpson's index**	**Shannon**
Tick genera	58			
*Amblyomma*	8	9.47 [5.20, 13.74]	0.32 [0.09, 0.55]	1.07 [0.32, 1.82]
*Dermacentor*	10	9.03 [4.61, 13.45]	0.39 [0.15, 0.62]	1.28 [0.48, 2.09]
*Haemaphysalis*	40	14.18 [12.62, 15.74]	0.55 [0.48, 0.61]	1.81 [1.58, 2.05]
Tick stages (only *Haemaphysalis* spp.)	36			
Larva	9	12.87 [9.30, 16.44]	0.58 [0.43, 0.72]	1.80 [1.35, 2.26]
Nymph	15	12.99 [10.72, 15.25]	0.58 [0.49, 0.68]	1.85 [1.52, 2.18]
Female	7	18.21 [15.77, 20.64]	0.46 [0.24, 0.67]	1.56 [0.89, 2.23]
Male	5	17.65 [9.71, 25.58]	0.48 [0.07, 0.89]	1.85 [0.28, 3.43]
*Francisella persica*	58			
Positive	4	3.08 [1.04, 5.11]	0.03 [−0.01, 0.08]	0.13 [−0.01, 0.26]
Negative	54	13.35 [11.97, 14.73]	0.52 [0.46, 0.58]	1.73 [1.52, 1.95]
*Coxiella-*like endosymbiont	58			
Positive	26	13.63 [11.72, 15.54]	0.50 [0.41, 0.59]	1.65 [1.36, 1.94]
Negative	32	11.84 [9.65, 14.03]	0.48 [0.37, 0.58]	1.60 [1.24, 1.95]
*Rickettsia* infection	58			
Positive	9	9.58 [5.08, 14.08]	0.37 [0.20, 0.54]	1.10 [0.59, 1.61]
Negative	49	13.20 [11.66, 14.74]	0.51 [0.44, 0.58]	1.72 [1.47, 1.97]
**Categories**	**Number of group**	**Group comparison (*p*-value)**		
Tick genera^*^	3	**0.01**	0.08	0.07
*Amblyomma* vs. Haemaphysalis^**^	2	**0.02**	**0.04**	**0.04**
*Dermacentor* vs. Haemaphysalis^**^	2	**0.02**	0.2	0.1
Tick stages^*^	4	**0.03**	0.6	0.9
Female vs Nymph^**^	2	**0.004**	0.2	0.5
Female vs. Larva^**^	2	**0.02**	0.2	0.4
*Francisella persica* infection^**^	2	**0.001**	**0.001**	**0.0009**
*Coxiella*-like endosymbiont^**^	2	0.3	1.0	0.9
*Rickettsia* infection^**^	2	0.06	0.08	**0.03**

*Statistical analysis used;*

(*), Kruskal–Wallis for across all groups comparison;

(**), Mann–Whitney U test for two groups comparison numbers in bold indicate statistical significance.

**Figure 5:**
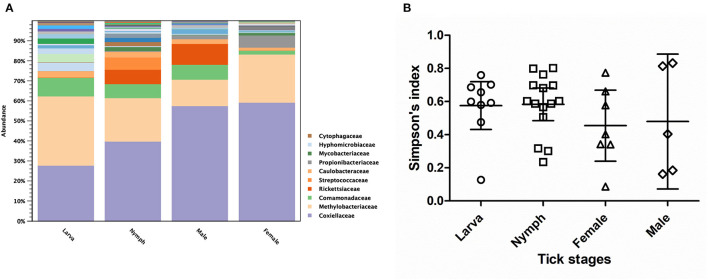
Taxonomic diversity and relative abundance at the family level of bacterial community **(A)** and scatter plots of alpha diversity measure (Simpson's index) **(B)** in three developmental stages of *Haemaphysalis* spp. ticks. The solid lines show mean and 95% Confidence Interval of alpha diversity for each group. Color legend for each family was indicated next to the bar graph.

As three *Haemaphysalis* spp. life stages (Larva = 9, Nymph = 15, Male = 6, Female = 10) were included in NGS, this genus was selected for testing the bacterial community difference among tick life stages (Figures 5A,B). Alpha diversity estimates (Simpson's index and Shannon entropy) showed that while the overall bacterial profiles among life stages were not significantly different with regard to bacterial taxa present, the number of observed OTUs among life stages was significantly different (Table 5; Figure 5B). Likewise, the beta diversity analysis using distance-based non-parametric test showed no significant difference across *Haemaphysalis* spp. life stages by the generalized UniFrac distance (df = 3, pseudo-*F* = 0.905, *p*-value = 0.5638). Comparing Coxiellaceae abundance across life stages, this group comprised 57–59% abundance of the total bacteria community in male and female ticks, 40% in nymphs, and 28% in larvae (Figure 5A).

## Discussion

In this study, we examined the bacterial community profile and co-infection of questing ticks collected by dragging from Khao Yai National Park using metagenomics. The majority of ticks collected were in the larval stage; however, adults and nymphs were also collected and identified as *Haemaphysalis* spp., *Amblyomma* spp., and *Dermacentor* spp. The greater number of *Haemaphysalis* spp. found in comparison to other genera may be due to the collection method used (dragging). The prevalence of *Rickettsia* spp. in ticks collected in Khao Yai National Park in this study was lower than in a previous report that found 30% of *Amblyomma testudinarium* positive for *Rickettsia* spp. and approximately 17% positive in *Haemaphysalis* spp. (44). However, they did not detect other bacteria such as *Borrelia* spp., *Francisella* spp., or the common symbiont *Wolbachia* spp. In this study, we detected *Rickettsia* DNA in 18% and 8% in *Amblyomma* spp. (*n* = 44) and *Haemaphysalis* spp. (*n* = 331) respectively, along with other bacteria such as *Anaplasma* spp., *Ehrlichia* spp., and *Borrelia* spp. at lower prevalence rates. We did not detect *Wolbachia* spp. in any of the ticks tested but found other bacterial endosymbionts, namely *Rickettsia* spp., *Francisella* spp., and *Coxiella* spp., using metagenomics and real-time PCR assay. Sequence analyses of *Coxiella* spp. and *Francisella* spp. detected by NGS confirmed the detection of these bacterial endosymbionts in examined ticks (Supplementary Figure 1). *Rickettsia* spp. that were detected by NGS but were not confirmed by subsequent qPCR and PCR assays are suspected to be a *Rickettsia* endosymbiont.

Co-infection of pathogenic bacteria occurred in a small number of ticks (*n* = 3), with the majority of co-infections between *Rickettsia* spp. and the Anaplasmataceae family. High prevalence of *Rickettsia* spp. in domestic animals and their ectoparasites, especially fleas, in Thailand has been recognized (15, 16, 45). Less than 10% of *Rickettsia* spp. was found in ticks collected from animals (45). However, some studies found infection rates as high as 24% for *Rickettsia* spp. and 32% for *Anaplasma* spp. in adult ticks collected from under leaves along animal trails across the country (46). The finding of co-infections between *Rickettsia* spp. and other bacteria in female ticks in this study was likely due to the ability of the bacteria to be maintained in ticks through transovarial and transstadial transmission (47–49). As females have already taken two bloodmeals during their development from larva to adult, the probability for *Rickettsia*-infected or Anaplasmataceae-infected ticks acquiring additional bacteria during feeding would be high (50–52). Our study also revealed the increased infection rate in adult ticks compared with larvae or nymphs. Similar findings were reported for an increasing *A. phagocytophilum* infection in adult ticks, especially in females, in Hanover, Germany (53). The study also reported that the co-infection between *A. phagocytophilum* and *Rickettsia* spp. was higher in females (5.2%) than in males (2.4%) or nymphs (1.6%).

A similar study was conducted by Nooroong et al. (46), in which ticks were collected under leaves along animal trails across Thailand were screened for bacterial pathogens. They found co-infections between *Rickettsia* spp. and *Anaplasma* spp. This co-infection was also observed in adult *A. testudinarium* ticks carrying the *Coxiella*-like endosymbiont in Nakhon Nayok, the same province where Khao Yai National Park is located. In Nooroong et al. (46), only adult ticks were collected and a 5.97% co-infection rate between *Rickettsia* spp. and *Anaplasma* spp., as well as a total infection rate of 35.8% for *Rickettsia*, was reported in the location near our study. This higher infection rate is likely due to the fact that Nooroong et al. (46) focused on the adult stage and did not include data from immature ticks. While all tick life stages collected by dragging were included in our study, the majority of ticks collected were larvae. Several previous studies in Thailand have primarily focused on ticks collected from animals; therefore, true co-infection status is inconclusive as pathogens may have been from the host animal. Our study focuses on co-infections in questing ticks actively seeking hosts, which directly represents the human risk of encountering ticks that can transmit multiple pathogens simultaneously. The co-infections investigated in previous studies were mostly among pathogenic bacteria and/or with bacterial endosymbionts (54), or among bacteria and protozoa such as *Coxiella* and *Babesia* spp. in *H. bispinosa* (55), or even co-infection with two to four pathogens of Anaplasmataceae, *Babesia* spp., and *Hepatozoon* spp. in *Rhipicephalus sanguineus* sensu lato collected from dogs in Bangkok, Thailand (56). Co-infection with more than three microorganisms was also reported in questing *Ixodes scapularis* ticks in Wisconsin, USA (29). Additionally, another study reported by Moutailler et al. (57) found up to 45% of questing *I. ricinus* ticks were co-infected with five to eight different pathogens including bacteria (*Borrelia* spp.), parasites, viruses, and endosymbionts. No significant interactions between endosymbionts and pathogens were found but there was a significant association between two *Borrelia* species: *B. garinii* and *B. afzelii*. Our results show that *Ehrlichia* infection in ticks harboring *Coxiella*-like endosymbiont was significantly higher than those without, suggesting that there might be some kind of relationship between *Ehrlichia* spp. and the *Coxiella*-like endosymbiont. However, we found no other association between Anaplasmataceae (*Ehrlichia* spp. and *Anaplasma* spp.) or *Rickettsia* spp. infection with the *Coxiella*-like endosymbiont. The data observed in this study implied that there was no negative or competitive effect of the *Coxiella*-like endosymbiont on the maintenance or existence of other bacteria in tick hosts. Endosymbionts relate to their host and pathogenic bacteria in many ways, such as providing nutrition lacking in blood meal, and sometimes possess an obligate relationship with the host (58, 59). However, other endosymbionts may interfere with the transmission to vertebrate hosts as was shown in *R. rickettsii* and *R. peacockii* in *D. andersoni* (60, 61). Another example of transmission interference can be seen in the salivary glands of *Amblyomma* spp., where a *Coxiella*-related symbiont impairs the transmission of *E. chaffeensis* (62). Alteration of the bacterial microbiome can also interfere with the colonization of pathogenic bacteria as seen in *B. burgdorferi* by modulation of the host immune response (63), which indicates that gut microbiota in ticks also plays a role in pathogen colonization in the gut lumen (22, 63).

Bacterial profiles using metagenomics used in this study showed a few dominant bacterial taxa harbored by questing ticks collected in Khao Yai National Park. Three main bacteria taxa were found in each tick genus—*Coxiella* spp. in *Haemaphysalis* spp., *Rickettsia* spp. in *Amblyomma* spp., and *Francisella* spp. in *Dermacentor* spp., making up the majority of bacterial taxa in ticks with relative abundance ranged from 40 to 80%. Our findings were consistent with other studies reporting that hard tick microbiomes are dominated by a small number of bacterial species, most of which are endosymbionts (20, 64). For example, *Coxiella* spp. was the main taxon found with a relative prevalence of 89.5% in *Rhipicephalus turanicus* (65), 89–100% for *A. americanum* (66–68), 98.2% in the female ovaries of *Rhipicephalus* (*Boophilus*) *microplus* (69), and 39.2% in *Haemaphysalis* spp. collected from animals in Malaysia (64). *Coxiella* spp. is a ubiquitous bacterium found in many tick species and maintained through transovarial transmission, as it was shown to pass on to their eggs and larvae from adult laboratory-reared *R. sanguineus* ticks (65, 70). Other bacterial endosymbionts detected in our study were *Rickettsia* spp., and depending on the tick species, *Rickettsia* spp. could represent up to 83% of the bacterial community, as observed in *Amblyomma* ticks in our study, while it was found to be less prevalent in the other two genera (*Haemaphysalis* and *Dermacentor*). A *Francisella*-like endosymbiont was another taxon we detected in high prevalence along with *Methylobacteria* in *Dermacentor* spp. The same finding was previously reported by several groups in which two *Dermacentor* species were dominated by three core bacterial taxa: *Francisella, Sphinogomonas*, and *Methylobacterium* (61, 71, 72). In addition to *Dermacentor* spp., *A. maculatum* was reported to harbor a *Francisella*-like endosymbiont at high abundance as well (73, 74). Our study also found that bacterial community composition varied significantly among tick genera, especially between *Amblyomma* and *Haemaphysalis* ticks. However, the species richness and diversity slightly decreased during development in *Haemaphysalis* spp. Other studies found that microbiome richness and diversity significantly decreased during development and varied greatly among species (72, 75). In one study, the obvious difference was on core OTUs of endosymbiont bacteria among tick species where *Dermacentor* spp. was dominated by *Francisella* spp., while *H. leporispalustris* and *I. pacificus* had *Coxiella* spp. and *Rickettsia* spp. as dominant bacterial species, respectively (72). Differences in reports might be from the techniques used to study the microbiome, geography, and natural vs. laboratory-reared tick populations (22, 76–78). However, the striking similarity among these studies is the difference in bacterial endosymbionts among tick genera, suggesting that there could be a competition among symbionts within tick genera. As it was reported in *R. turanicus, Coxiella* was the primary symbiont and *Rickettsia* was the secondary, having lesser relative abundance (65). Other studies discovered competition between *Rickettsia* species in *Dermacentor* spp. in which one species prevented another from transovarial transmission (79, 80).

Understanding the microbiome composition in ticks of different species, their vector capacities, as well as the role of bacterial endosymbionts on tick physiology, including their influence on pathogen transmission, may provide insight into vector control to prevent human infection and the emergence of tick-borne diseases in the future. This study provides important information on bacterial community composition and co-infection rates in questing ticks in Thailand with implications for animal and human health.

## Data Availability Statement

The datasets presented in this study can be found in online repositories. The names of the repository/repositories and accession number(s) can be found below: GenBank accession number(s) OK161258-61, OK161265-68, OK161272-79, OK161279-84, and OK180561-611. The SRA BioProject ID PRJNA766341.

## Ethics Statement

The animal study was reviewed and approved by AFRIMS Institutional Animal Care and Use Committee (IACUC), U.S. Army Medical Directorate-Armed Forces Research Institute of Medical Sciences.

## Author Contributions

RT and SC conceived and designed the experiments. JS, NC, NY, SP, and BT performed the experiments. SP, BT, NC, WT, KS, and SC conducted field works. RT and JS analyzed the data. RT wrote/revised the manuscript. BP-S and PM reviewed the manuscript. All authors contributed to the article and approved the submitted version.

## Funding

This work was funded by the Armed Forces Health Surveillance Division (AFHSD) Global Emerging Infections Surveillance Branch (GEIS), ProMIS ID # P0128_20_AF_04.05.

## Author Disclaimer

Material has been reviewed by the Walter Reed Army Institute of Research. There is no objection to its presentation and/or publication. The opinions or assertions contained herein are the private views of the author, and are not to be construed as official, or as reflecting true views of the Department of the Army or the Department of Defense. Research was conducted under an approved animal use protocol as part of an AAALAC International-accredited facility in compliance with the Animal Welfare Act and other federal statutes and regulations relating to animals and experiments involving animals and adheres to principles stated in the Guide for the Care and Use of Laboratory Animals, NRC Publication, 2011 edition.

## Conflict of Interest

The authors declare that the research was conducted in the absence of any commercial or financial relationships that could be construed as a potential conflict of interest.

## Publisher's Note

All claims expressed in this article are solely those of the authors and do not necessarily represent those of their affiliated organizations, or those of the publisher, the editors and the reviewers. Any product that may be evaluated in this article, or claim that may be made by its manufacturer, is not guaranteed or endorsed by the publisher.
