# Supplementary material for: The Bacterial Community in Questing Ticks From Khao Yai National Park in Thailand
Source: Front Vet Sci. 2021 Nov 22;8:764763. doi: 10.3389/fvets.2021.764763 (PMC8645651; doi:10.3389/fvets.2021.764763)
Supplement: Supplementary file 1 [file Table_1.pdf]

Table S1. Conditions, primer&probe sequences, and references for real-time PCR, and PCR procedures for sequencing in confirmatory assays.

Pathogen	Target Gene	Product size (bp)	Assay type(s)	Primer & probe name	Primer & Probe sequence (5'-->3')	Probes Concentrati	$MgCl_2$ Concentration	Annealing Temperature	Reference
Anaplasma spp. *Ehrlichia* spp. *Neoehrlichia* spp. *Neorickettsia* spp.	16S	345	PCR and sequencing	AE spp-16S rDNA-F	GGTACCYACAGAAGAAGTCC	0.2	1.75	52	(Pereira et al., 2018)
				AE spp-16S rDNA-R	TAGCACTCATCGTTTACAGC	0.2			
Anaplasma spp. *Ehrlichia* spp.	groEL	600	PCR and sequencing	AE-groEL-F	ACTGATGGTATGCARTTTGAYCG	0.2	1.75	52	(Pereira et al., 2018)
				AE-groEL-R	TCTTTRCGTTCYTTMACYTCAACTTC	0.2			
Borrelia spp.	16S	148	Real-time PCR	Bor 16S 3F	AGCCTTTAAAGCTTCGCTTGTAG	0.2	3	60	(Parola et al., 2011)
				Bor 16S 3R	GCCTCCCGTAGGAGTCTGG	0.2			
				Bor 16S 3P	6FAM-CCGGCCTGAGAGGGTGAACGG-BHQ1	0.2			
Borrelia spp.	16S	1427	nested PCR and sequencing	B1	CAG TGC GTC TTA AGC ATG C	0.5	1.0	56	(Park et al., 2004)
				B8	CCT TAA ATA CCT TCC TCC C	0.5			
	16S (Nested)	714		B3	GCA GCT AAG AAT CTT CCG CAA TGG	0.5	1.0	59	
				B6	CAA CCA TGC AGC ACC TGT ATA T	0.5			
	flaB	819	nested PCR and sequencing	FLA120F	AGAATTAATMGHGCWTCTGATGATG	0.3	1.5	54	(Scoles et al., 2001)
				Fla920R	TGCYACAAYHTCATCTGTCATT	0.3			
	flaB (Nested)	354		FlaLS	AACAGCTGAAGAGCTTGGAATG	0.3	1.5	58	(Barbour et al., 1996)
				FlaRS	CTTTGATCACTTATCATTCTAATAGC	0.3			
Coxiella burnetii	IS1111	294	Real-time PCR	Cox-F	GTCTTAAGGTGGGCTGCGTG	0.3	3	60	(Klee et al., 2006)
				Cox-R	CCCCGAATCTCATTGATCAGC	0.3			
				Cox-TM	FAM- AGCGAACCATTGGTATCGGACGTTTATGG-TAMRA	0.2			
Coxeilla spp.	16S	500	Real-time PCR (SYBR green) and sequencing	Cox sp434f	CCTTTTGAGCGTTGACGTTA	0.2	2.5	63	(Lalzar et al., 2012)
				Cox sp1004r	CCAAAGGCACCAAGTCATTT	0.2			
Francisella spp.	*tip* A	484	PCR and sequencing	tpiA-F	TGG TCA TTC TGA GAG AAG ATC	0.2		52	(Takhampunya et al., 2017)
				tpiA-R	CGT ACA TAT CTT GTT TGC TTG	0.2			
Francisella spp.	16S	1128	PCR and sequencing	Fr153F0.1	GCCCATTTGAGGGGGATACC	0.2	2.5	60	(Barns et al., 2005)
				Fr1281R0.1	GGACTAAGAGTACCTTTTTGAGT	0.2			
Rickettsia spp.	17 kDa	111	Real-time PCR	R17K 238R	CCT ACA CCT ACT CCV ACA AG	0.1	3	60	(Wright et al., 2011)
				R17K128F2	GGG CGG TAT GAA YAA ACA AG	0.1			
				R17Kprobe	FAM -CCG AAT TGA GAA CCA AGT AAT GC- TAMRA	0.1			
Rickettsia spp.	gltA	850	PCR and sequencing	RpCS.409d	CCT ATG GCT ATT ATG CTT GC	0.2	2.5	55	(Chmielewski et al., 2009
				RpCS.1258n	ATT GCA AAA AGT ACA GTG AAC A	0.2			
