# Supplementary material for: The Bacterial Community in Questing Ticks From Khao Yai National Park in Thailand
Source: Front Vet Sci. 2021 Nov 22;8:764763. doi: 10.3389/fvets.2021.764763 (PMC8645651; doi:10.3389/fvets.2021.764763)
Supplement: Supplementary file 2 [file Table_2.pdf]

Table S2. Ticks selected for NGS experiment in more detail.

	Number of ticks selected for NGS experiment (Number of ticks that passed quality-filter)				
**Tick species/stages**	**Larvae**	**Nymphs**	**Males**	**Females**	**Selected for NGS**
Amblyomma spp.	5 (5)	2 (2)	0	0	7 (7)
Amblyomma testudinarium	0	0	1 (0)	3 (1)	4 (1)
Dermacentor spp.	5 (5)	0	0	0	5 (5)
Dermacentor auratus	0	0	3 (2)	3 (2)	6 (4)
Dermacentor steini	0	0	1 (0)	2 (1)	3 (1)
Haemaphysalis spp.	10 (9)	20 (15)	0	0	30 (24)
Haemaphysalis lagrangei	0	0	3 (1)	3 (2)	6 (3)
Haemaphysalis longicornis	0	0	3 (2)	3 (3)	6 (5)
Haemaphysalis obesa	0	0	3 (1)	3 (2)	6 (3)
Haemaphysalis papuana	0	0	0	3 (1)	3 (1)
Haemaphysalis shimoga	0	0	3 (2)	3 (2)	6 (4)
**Selected for NGS**	**20 (19)**	**22 (17)**	**17 (8)**	**23 (14)**	**82 (58)**
