# Supplementary material for: The Bacterial Community in Questing Ticks From Khao Yai National Park in Thailand
Source: Front Vet Sci. 2021 Nov 22;8:764763. doi: 10.3389/fvets.2021.764763 (PMC8645651; doi:10.3389/fvets.2021.764763)

Figure S1. Phylogenetic analysis of pathogens detected from NGS and conventional Sanger sequencing (red letters). All maximum likelihood trees were created using the best fit model of nucleotide substitution in the MEGA 6 program with bootstrapping (1000 replicates).

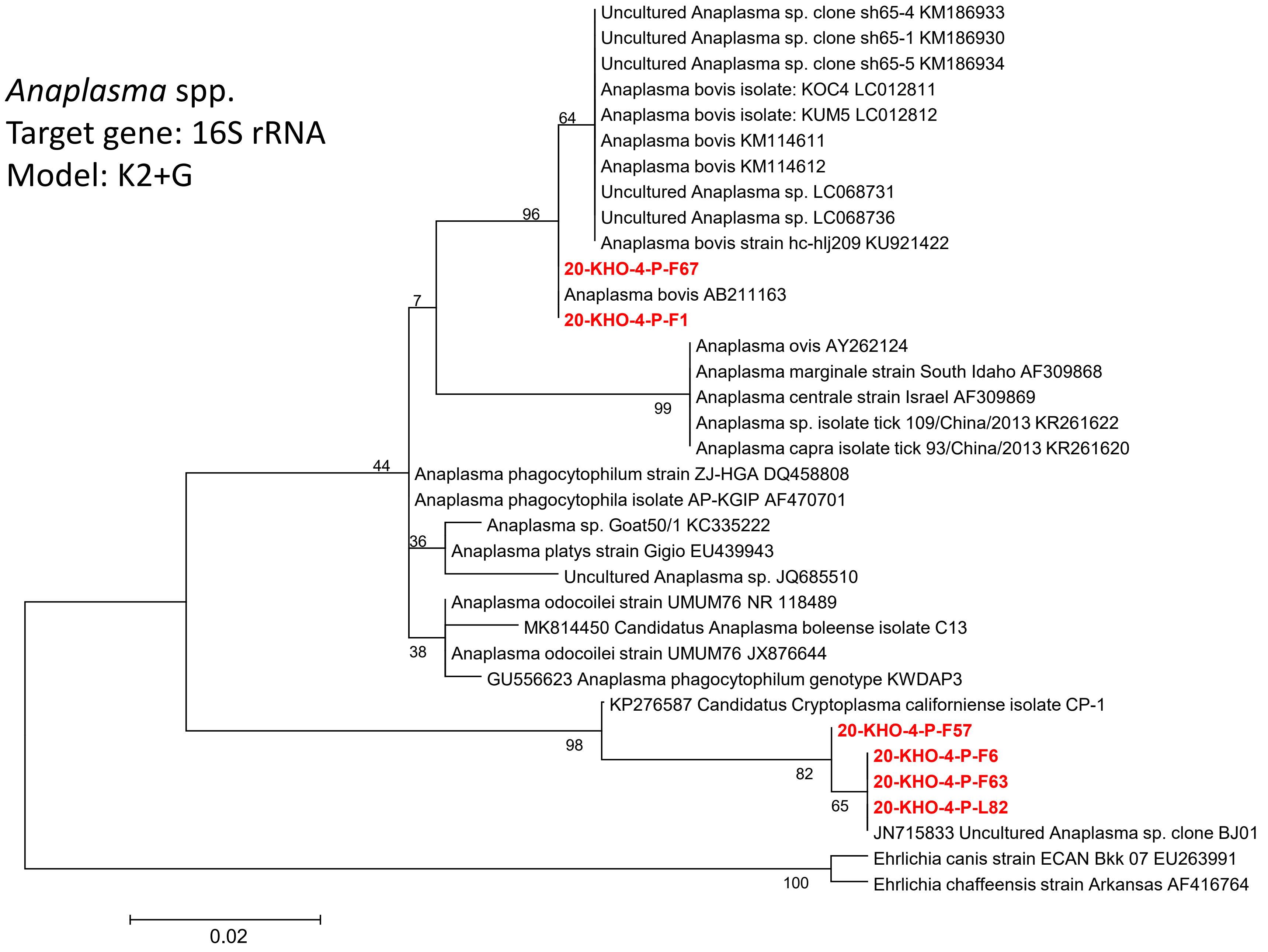

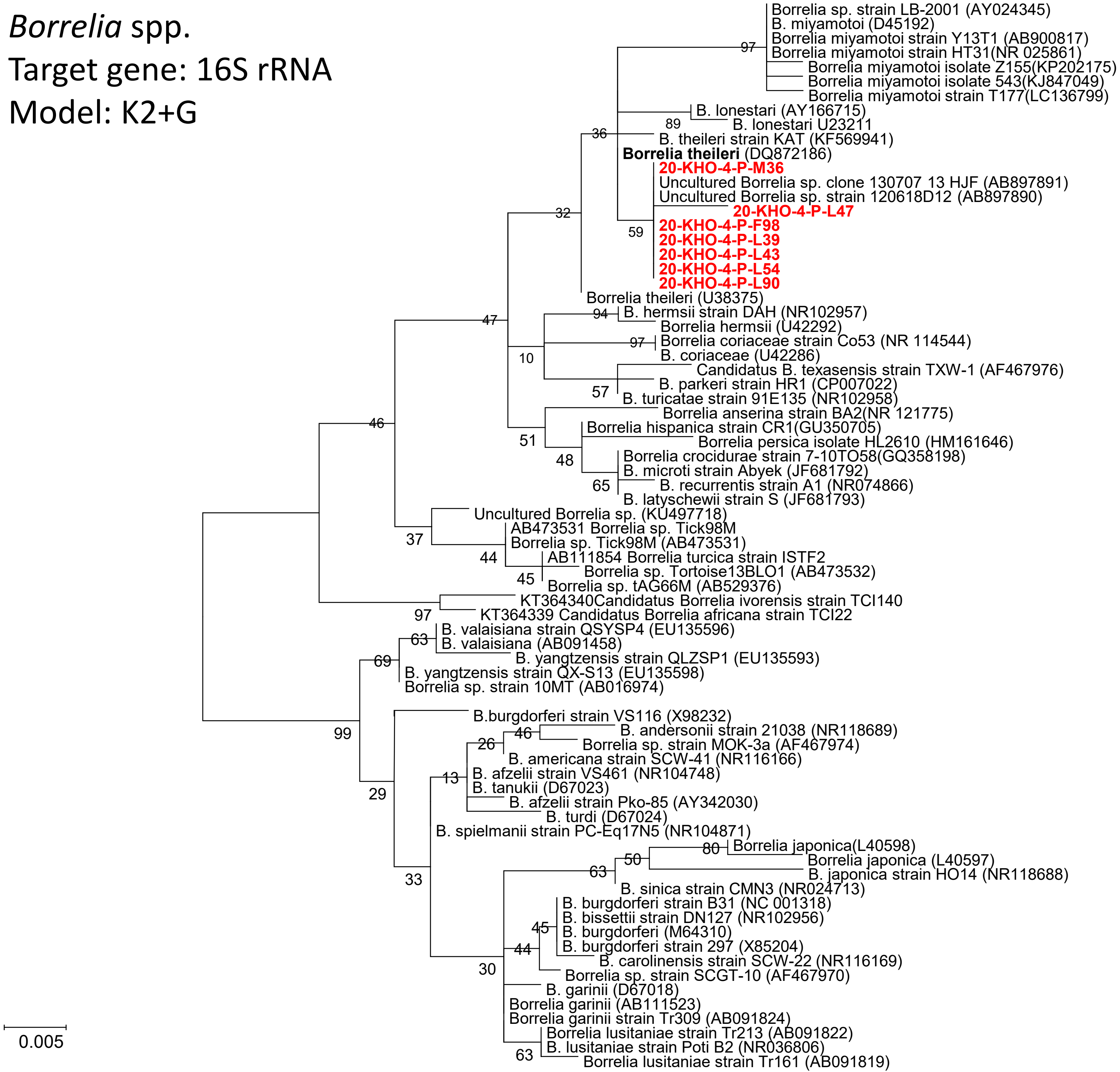
Borrelia spp.
Target gene: 16S rRNA
Model: K2+G
Borrelia sp. strain LB-2001 (AY024345)
B. miyamotoi (D45192)
Borrelia miyamotoi strain Y13T1 (AB900817)
Borrelia miyamotoi strain HT31(NR 025861)
Borrelia miyamotoi isolate Z155(KP202175)
Borrelia miyamotoi isolate 543(KJ847049)
Borrelia miyamotoi strain T177(LC136799)
B. lonestari (AY166715)
B. lonestari U23211
B. theileri strain KAT (KF569941)
Borrelia theileri (DQ872186)
20-KHO-4-P-M36
Uncultured Borrelia sp. clone 130707 13 HJF (AB897891)
Uncultured Borrelia sp. strain 120618D12 (AB897890)
20-KHO-4-P-L47
20-KHO-4-P-F98
20-KHO-4-P-L39
20-KHO-4-P-L43
20-KHO-4-P-L54
20-KHO-4-P-L90
Borrelia theileri (U38375)
B. hermsii strain DAH (NR102957)
Borrelia hermsii (U42292)
Borrelia coriaceae strain Co53 (NR 114544)
B. coriaceae (U42286)
Candidatus B. texasensis strain TXW-1 (AF467976)
B. parkeri strain HR1 (CP007022)
B. turicatae strain 91E135 (NR102958)
Borrelia anserina strain BA2(NR 121775)
Borrelia hispanica strain CR1(GU350705)
Borrelia persica isolate HL2610 (HM161646)
Borrelia crocidurae strain 7-10TO58(GQ358198)
B. microti strain Abyek (JF681792)
B. recurrentis strain A1 (NR074866)
B. latyschewii strain S (JF681793)
Uncultured Borrelia sp. (KU497718)
AB473531 Borrelia sp. Tick98M
Borrelia sp. Tick98M (AB473531)
AB111854 Borrelia turcica strain ISTF2
Borrelia sp. Tortoise13BLO1 (AB473532)
Borrelia sp. tAG66M (AB529376)
KT364340Candidatus Borrelia ivorensis strain TCI140
KT364339 Candidatus Borrelia africana strain TCI22
B. valaisiana strain QSYSP4 (EU135596)
B. valaisiana (AB091458)
B. yangtzensis strain QLZSP1 (EU135593)
B. yangtzensis strain QX-S13 (EU135598)
Borrelia sp. strain 10MT (AB016974)
B.burgdorferi strain VS116 (X98232)
B. andersonii strain 21038 (NR118689)
Borrelia sp. strain MOK-3a (AF467974)
B. americana strain SCW-41 (NR116166)
B. afzelii strain VS461 (NR104748)
B. tanukii (D67023)
B. afzelii strain Pko-85 (AY342030)
B. turdi (D67024)
B. spielmanii strain PC-Eq17N5 (NR104871)
Borrelia japonica(L40598)
Borrelia japonica (L40597)
B. japonica strain HO14 (NR118688)
B. sinica strain CMN3 (NR024713)
B. burgdorferi strain B31 (NC 001318)
B. bissettii strain DN127 (NR102956)
B. burgdorferi (M64310)
B. burgdorferi strain 297 (X85204)
B. carolinensis strain SCW-22 (NR116169)
Borrelia sp. strain SCGT-10 (AF467970)
B. garinii (D67018)
Borrelia garinii (AB111523)
Borrelia garinii strain Tr309 (AB091824)
Borrelia lusitaniae strain Tr213 (AB091822)
B. lusitaniae strain Poti B2 (NR036806)
Borrelia lusitaniae strain Tr161 (AB091819)
0.005

Ehrlichia spp.

Target gene: *gro*EL

Model: T92+G

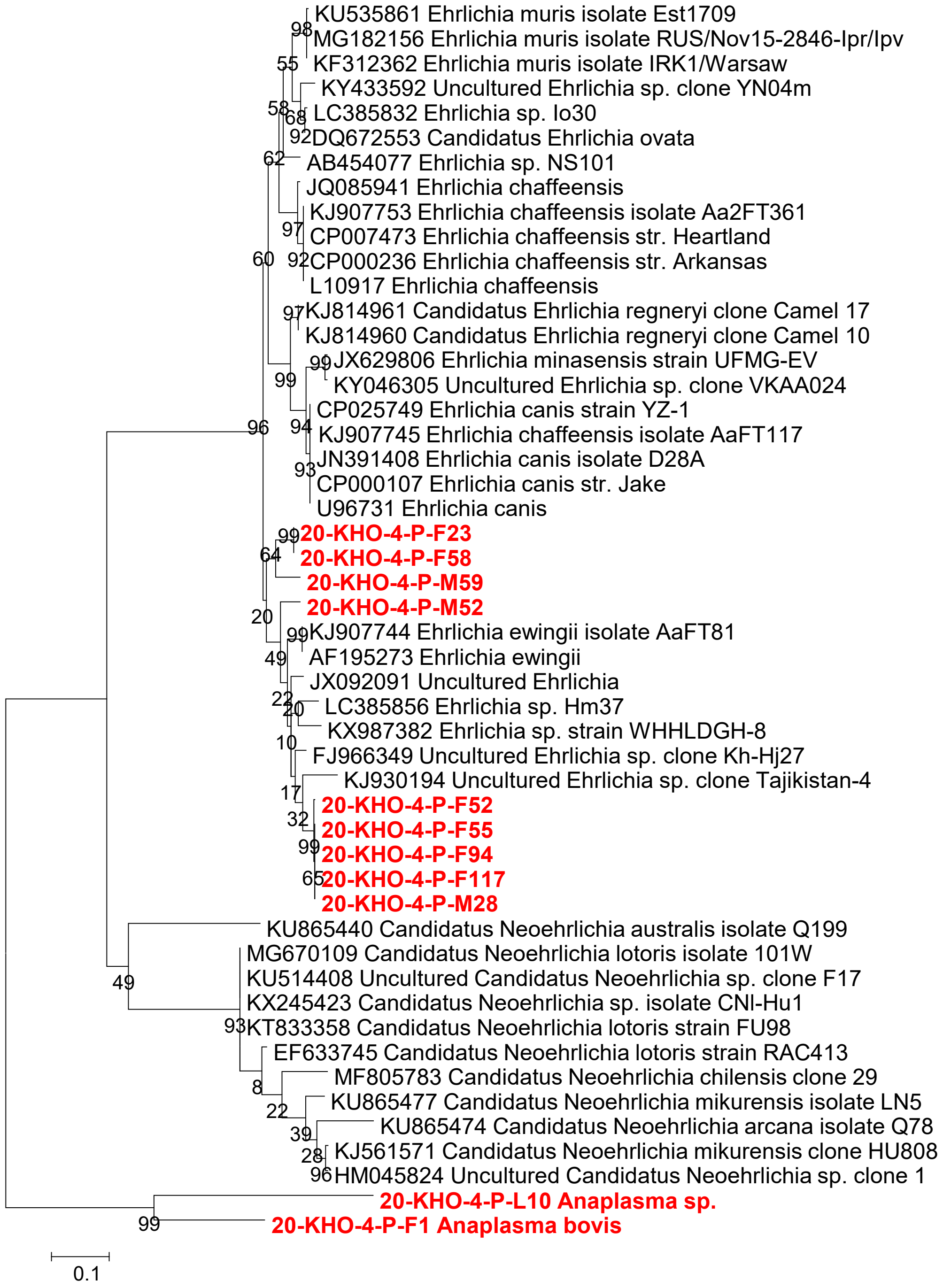

Anaplasmataceae
Target gene: *gro*EL
Model: T92+G

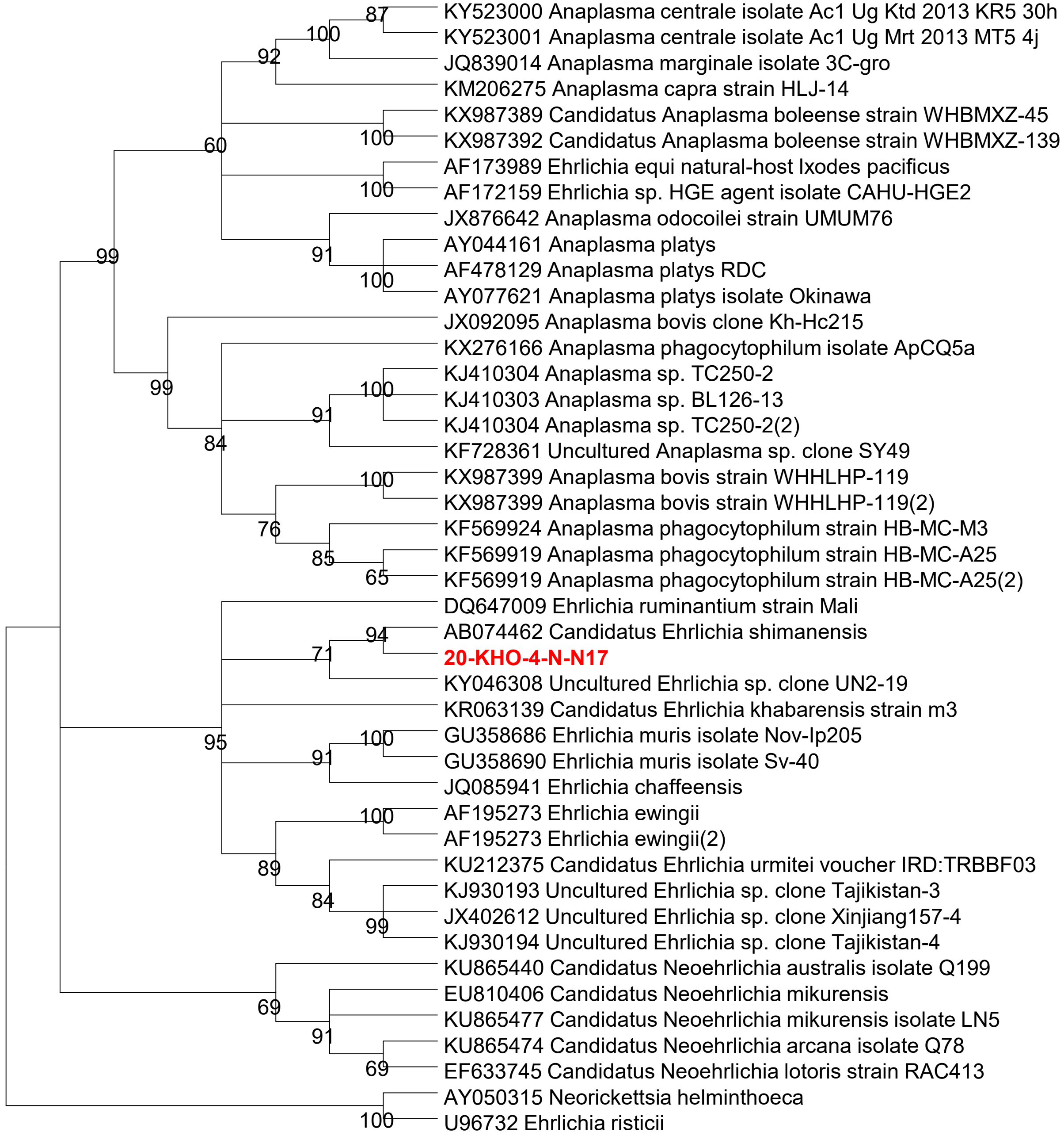

Francisella spp.
Target gene: *tpi*A
Model: T92+G

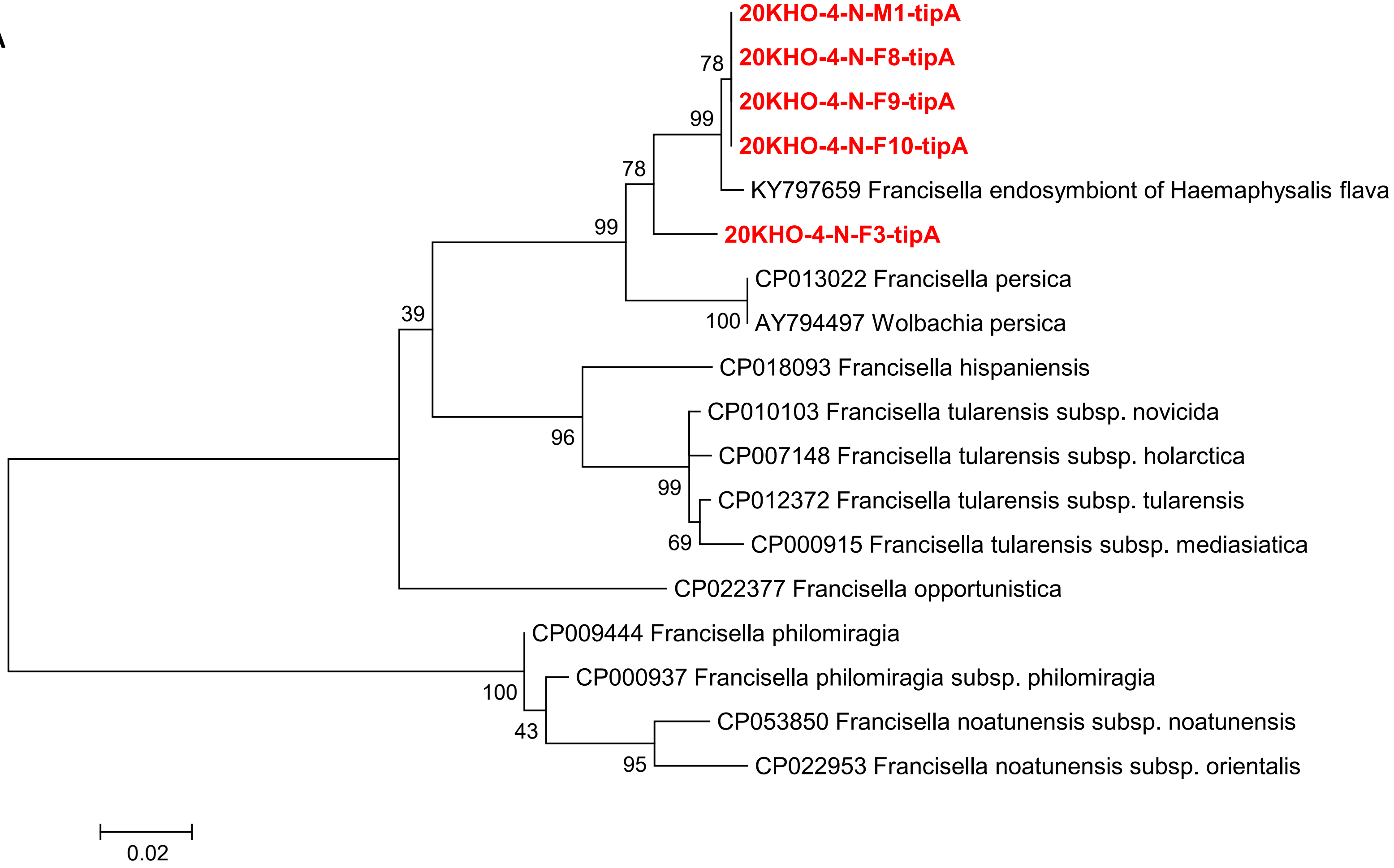

Francisella spp. and *Francisella*-like endosymbiont (FLE)
Target gene: 16S rRNA
Model: K2+G

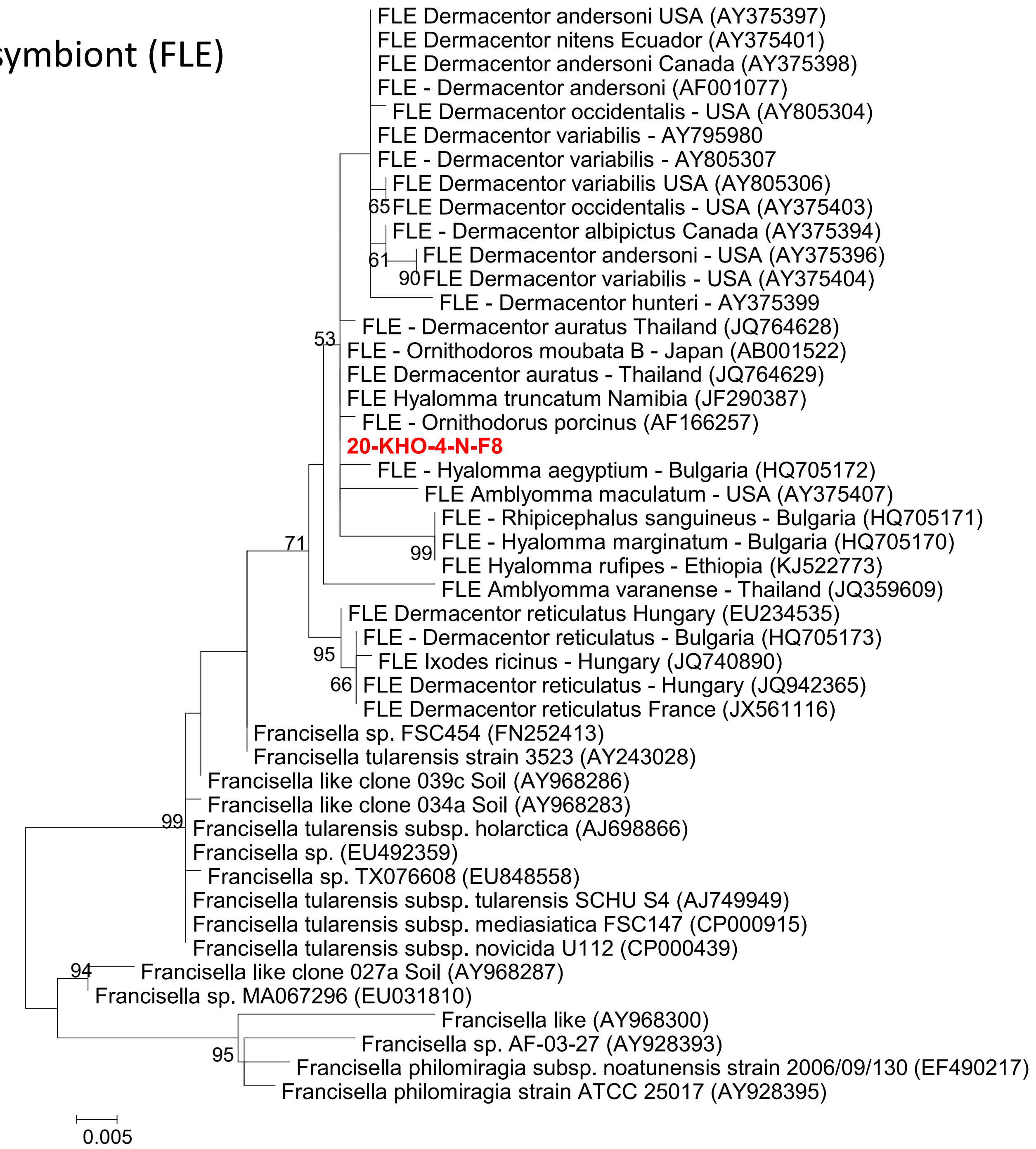

Coxiella endosymbiont
Target gene: 16S rRNA
Model: K2+G

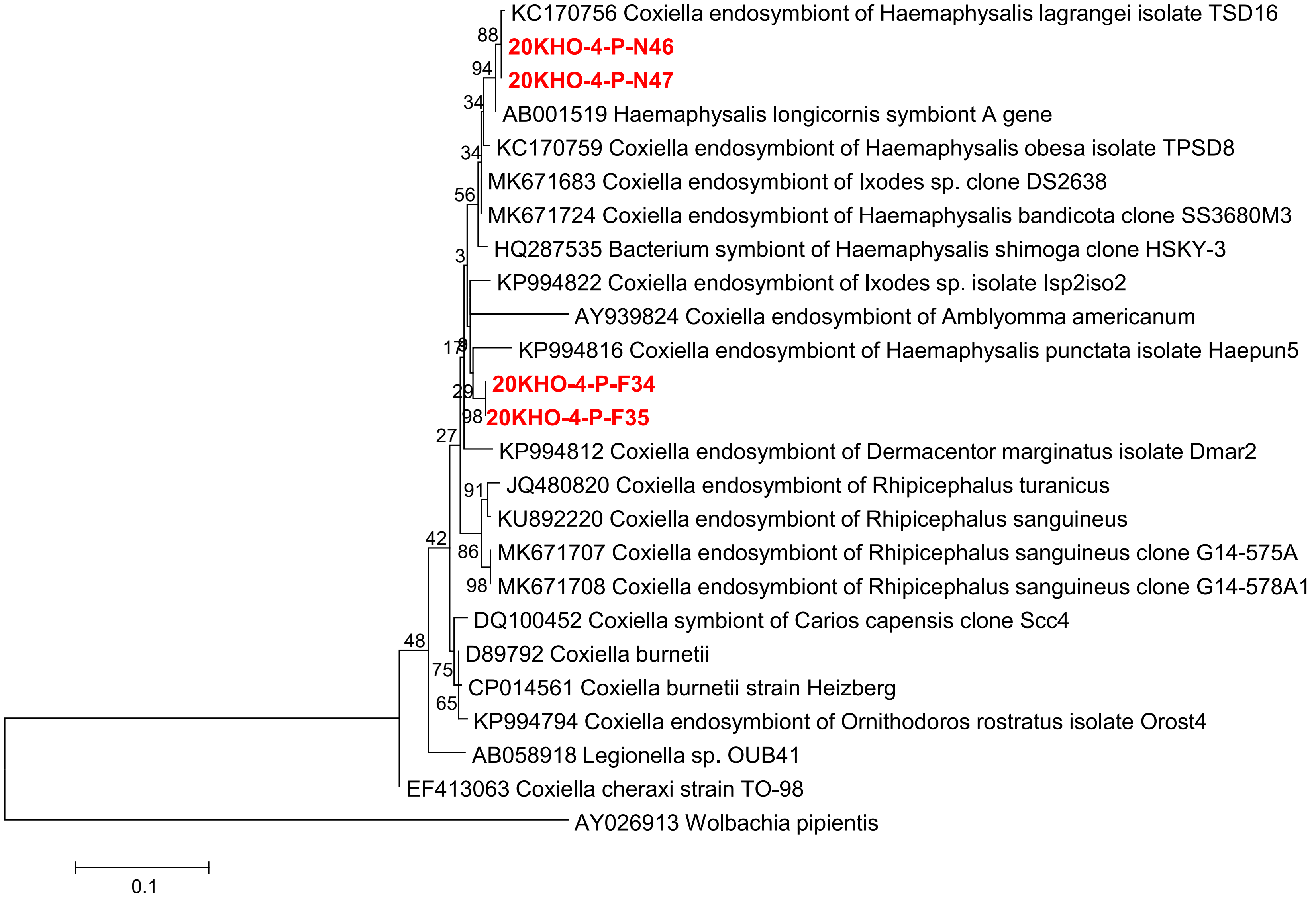

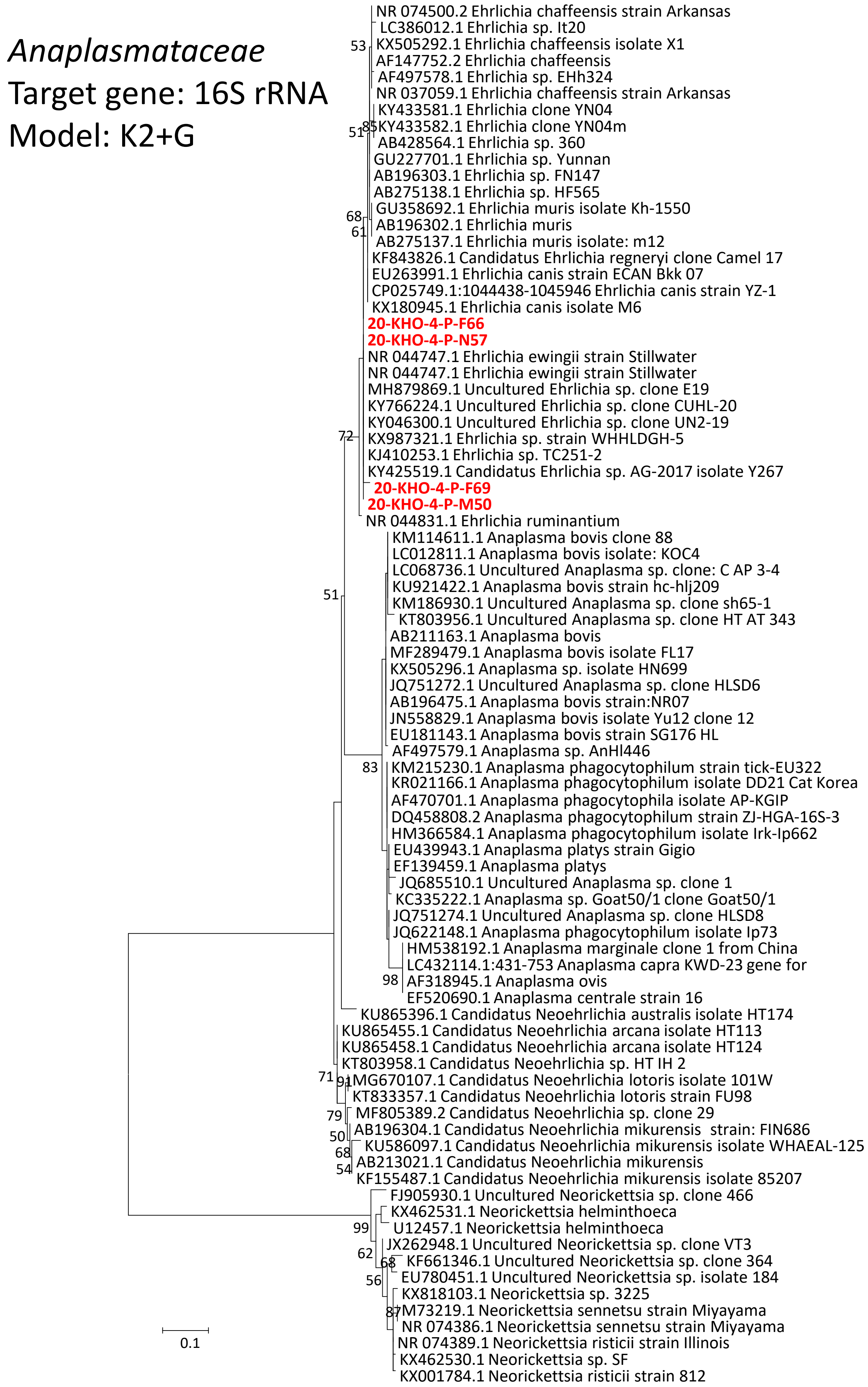
Anaplasmataceae
Target gene: 16S rRNA
Model: K2+G
NR 074500.2 Ehrlichia chaffeensis strain Arkansas
LC386012.1 Ehrlichia sp. It20
KX505292.1 Ehrlichia chaffeensis isolate X1
AF147752.2 Ehrlichia chaffeensis
AF497578.1 Ehrlichia sp. EHh324
NR 037059.1 Ehrlichia chaffeensis strain Arkansas
KY433581.1 Ehrlichia clone YN04
KY433582.1 Ehrlichia clone YN04m
AB428564.1 Ehrlichia sp. 360
GU227701.1 Ehrlichia sp. Yunnan
AB196303.1 Ehrlichia sp. FN147
AB275138.1 Ehrlichia sp. HF565
GU358692.1 Ehrlichia muris isolate Kh-1550
AB196302.1 Ehrlichia muris
AB275137.1 Ehrlichia muris isolate: m12
KF843826.1 Candidatus Ehrlichia regneryi clone Camel 17
EU263991.1 Ehrlichia canis strain ECAN Bkk 07
CP025749.1:1044438-1045946 Ehrlichia canis strain YZ-1
KX180945.1 Ehrlichia canis isolate M6
20-KHO-4-P-F66
20-KHO-4-P-N57
NR 044747.1 Ehrlichia ewingii strain Stillwater
NR 044747.1 Ehrlichia ewingii strain Stillwater
MH879869.1 Uncultured Ehrlichia sp. clone E19
KY766224.1 Uncultured Ehrlichia sp. clone CUHL-20
KY046300.1 Uncultured Ehrlichia sp. clone UN2-19
KX987321.1 Ehrlichia sp. strain WHHLDGH-5
KJ410253.1 Ehrlichia sp. TC251-2
KY425519.1 Candidatus Ehrlichia sp. AG-2017 isolate Y267
20-KHO-4-P-F69
20-KHO-4-P-M50
NR 044831.1 Ehrlichia ruminantium
KM114611.1 Anaplasma bovis clone 88
LC012811.1 Anaplasma bovis isolate: KOC4
LC068736.1 Uncultured Anaplasma sp. clone: C AP 3-4
KU921422.1 Anaplasma bovis strain hc-hlj209
KM186930.1 Uncultured Anaplasma sp. clone sh65-1
KT803956.1 Uncultured Anaplasma sp. clone HT AT 343
AB211163.1 Anaplasma bovis
MF289479.1 Anaplasma bovis isolate FL17
KX505296.1 Anaplasma sp. isolate HN699
JQ751272.1 Uncultured Anaplasma sp. clone HLSD6
AB196475.1 Anaplasma bovis strain:NR07
JN558829.1 Anaplasma bovis isolate Yu12 clone 12
EU181143.1 Anaplasma bovis strain SG176 HL
AF497579.1 Anaplasma sp. AnHl446
KM215230.1 Anaplasma phagocytophilum strain tick-EU322
KR021166.1 Anaplasma phagocytophilum isolate DD21 Cat Korea
AF470701.1 Anaplasma phagocytophila isolate AP-KGIP
DQ458808.2 Anaplasma phagocytophilum strain ZJ-HGA-16S-3
HM366584.1 Anaplasma phagocytophilum isolate Irk-Ip662
EU439943.1 Anaplasma platys strain Gigio
EF139459.1 Anaplasma platys
JQ685510.1 Uncultured Anaplasma sp. clone 1
KC335222.1 Anaplasma sp. Goat50/1 clone Goat50/1
JQ751274.1 Uncultured Anaplasma sp. clone HLSD8
JQ622148.1 Anaplasma phagocytophilum isolate Ip73
HM538192.1 Anaplasma marginale clone 1 from China
LC432114.1:431-753 Anaplasma capra KWD-23 gene for
AF318945.1 Anaplasma ovis
EF520690.1 Anaplasma centrale strain 16
KU865396.1 Candidatus Neoehrlichia australis isolate HT174
KU865455.1 Candidatus Neoehrlichia arcana isolate HT113
KU865458.1 Candidatus Neoehrlichia arcana isolate HT124
KT803958.1 Candidatus Neoehrlichia sp. HT IH 2
MG670107.1 Candidatus Neoehrlichia lotoris isolate 101W
KT833357.1 Candidatus Neoehrlichia lotoris strain FU98
MF805389.2 Candidatus Neoehrlichia sp. clone 29
AB196304.1 Candidatus Neoehrlichia mikurensis strain: FIN686
KU586097.1 Candidatus Neoehrlichia mikurensis isolate WHAEAL-125
AB213021.1 Candidatus Neoehrlichia mikurensis
KF155487.1 Candidatus Neoehrlichia mikurensis isolate 85207
FJ905930.1 Uncultured Neorickettsia sp. clone 466
KX462531.1 Neorickettsia helminthoeca
U12457.1 Neorickettsia helminthoeca
JX262948.1 Uncultured Neorickettsia sp. clone VT3
KF661346.1 Uncultured Neorickettsia sp. clone 364
EU780451.1 Uncultured Neorickettsia sp. isolate 184
KX818103.1 Neorickettsia sp. 3225
M73219.1 Neorickettsia sennetsu strain Miyayama
NR 074386.1 Neorickettsia sennetsu strain Miyayama
NR 074389.1 Neorickettsia risticii strain Illinois
KX462530.1 Neorickettsia sp. SF
KX001784.1 Neorickettsia risticii strain 812
0.1

Rickettsia spp.
Target gene: *glt*A
Model: T92+G

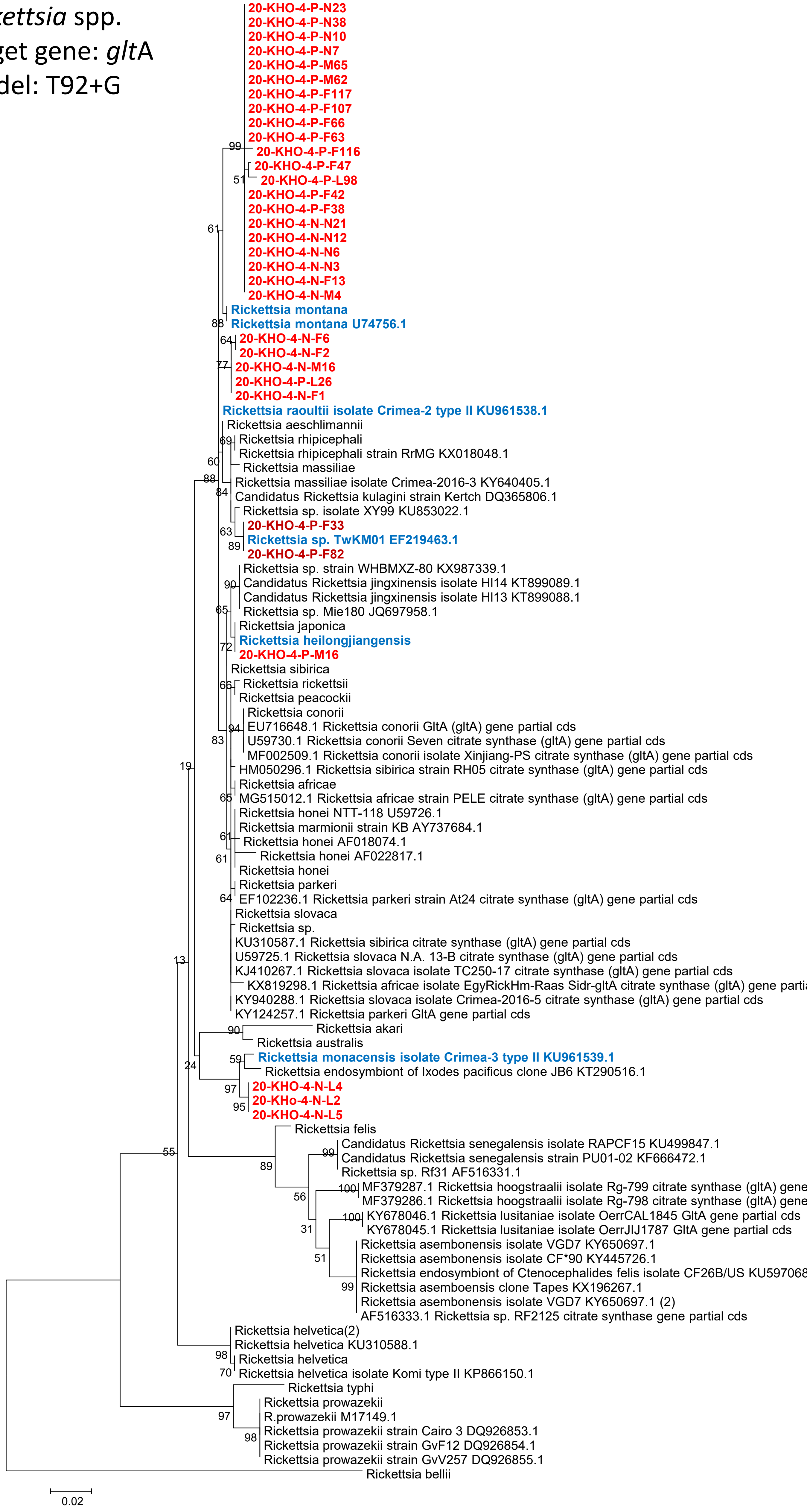

Supplement: Supplementary file 3 [file Data_Sheet_1.PDF]
