# Supplementary figures and images for: The Bacterial Community in Questing Ticks From Khao Yai National Park in Thailand
Source: Front Vet Sci. 2021 Nov 22;8:764763. doi: 10.3389/fvets.2021.764763 (PMC8645651; doi:10.3389/fvets.2021.764763)

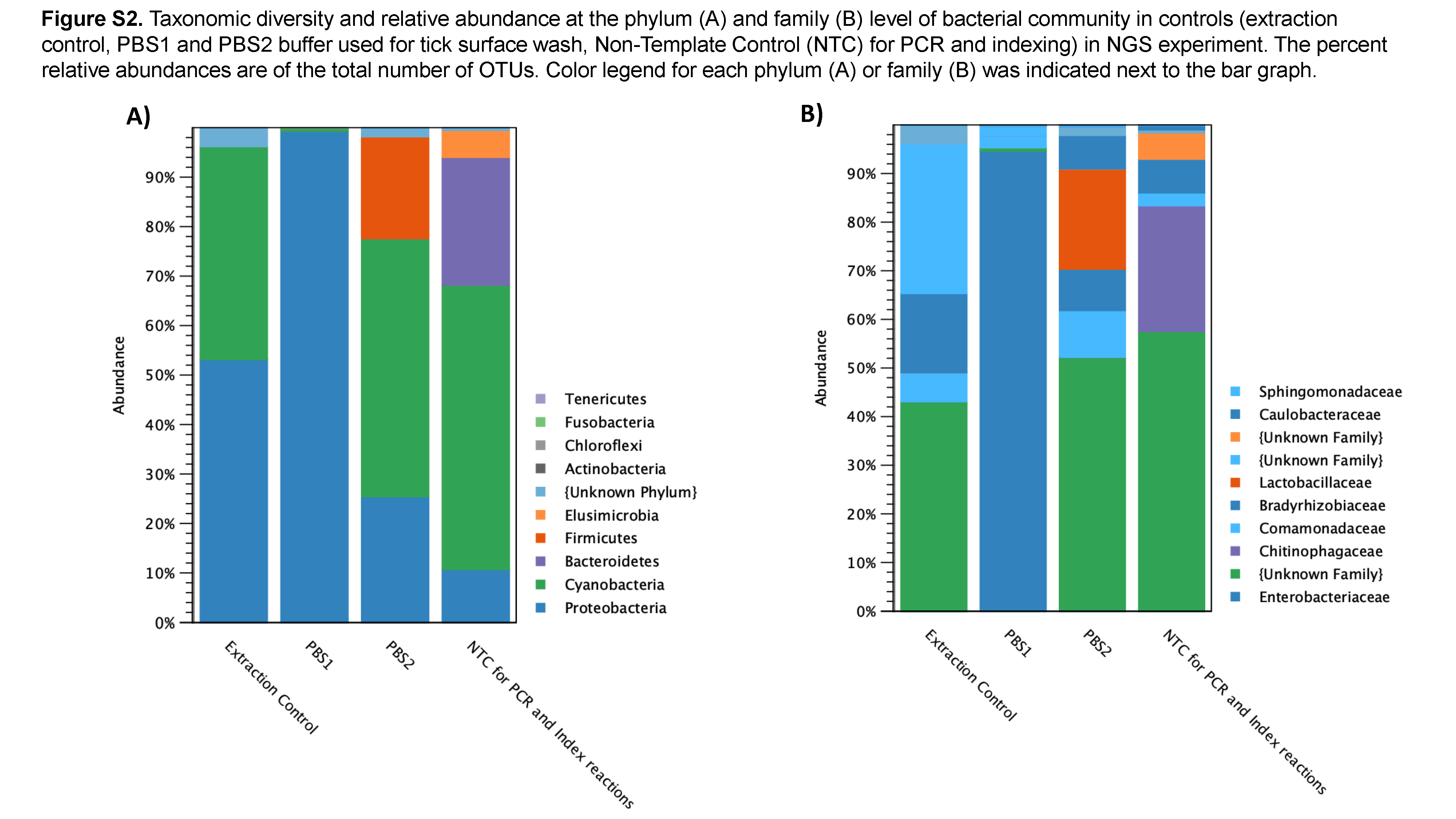

Supplement: Supplementary file 4 [file Image_1.TIFF]
